# Homopolymer tract organization in the human malarial parasite *Plasmodium falciparum* and related Apicomplexan parasites

**DOI:** 10.1186/1471-2164-15-848

**Published:** 2014-10-03

**Authors:** Karen Russell, Chia-Ho Cheng, Jeffrey W Bizzaro, Nadia Ponts, Richard D Emes, Karine Le Roch, Kenneth A Marx, Paul Horrocks

**Affiliations:** Institute for Science and Technology in Medicine, Keele University, Stoke-on-Trent, ST5 5BG Staffordshire, UK; Center for Intelligent Biomaterials, University of Massachusetts Lowell, Lowell, MA 01854 USA; Bioinformatics Organization Inc, Hudson, MA 01749 USA; National Institute for Agricultural Research (INRA), UR1264-Mycology and Food Safety (MycSA), CS20032, 33882 Villenave d’Ornon Cedex, France; School of Veterinary Medicine and Science, University of Nottingham, LE12 5RD Nottingham, Leicestershire, UK; Advanced Data Analysis Centre, University of Nottingham, Nottingham, UK; Department Cell Biology and Neuroscience, University of California, Riverside, CA 92521 USA; Hebrew SeniorLife, Institute for Aging Research, Boston, MA 02131 USA

**Keywords:** Poly dA.dT, Intergenic regions, Malaria, Nucleosome, Gene expression

## Abstract

**Background:**

Homopolymeric tracts, particularly poly dA.dT, are enriched within the intergenic sequences of eukaryotic genomes where they appear to act as intrinsic regulators of nucleosome positioning. A previous study of the incomplete genome of the human malarial parasite *Plasmodium falciparum* reports a higher than expected enrichment of poly dA.dT tracts, far above that anticipated even in this highly AT rich genome. Here we report an analysis of the relative frequency, length and spatial arrangement of homopolymer tracts for the complete *P. falciparum* genome, extending this analysis to twelve additional genomes of Apicomplexan parasites important to human and animal health. In addition, using nucleosome-positioning data available for *P. falciparum*, we explore the correlation of poly dA.dT tracts with nucleosome-positioning data over key expression landmarks within intergenic regions.

**Results:**

We describe three apparent lineage-specific patterns of homopolymeric tract organization within the intergenic regions of these Apicomplexan parasites. Moreover, a striking pattern of enrichment of overly long poly dA.dT tracts in the intergenic regions of *Plasmodium* spp. uniquely extends into protein coding sequences. There is a conserved spatial arrangement of poly dA.dT immediately flanking open reading frames and over predicted core promoter sites. These key landmarks are all relatively depleted in nucleosomes in *P. falciparum*, as would be expected for poly dA.dT acting as nucleosome exclusion sequences.

**Conclusions:**

Previous comparative studies of homopolymer tract organization emphasize evolutionary diversity; this is the first report of such an analysis within a single phylum. Our data provide insights into the evolution of homopolymeric tracts and the selective pressures at play in their maintenance and expansion.

**Electronic supplementary material:**

The online version of this article (doi:10.1186/1471-2164-15-848) contains supplementary material, which is available to authorized users.

## Background

Genome-wide surveys of homopolymer tract frequencies in eukaryotic genomes reveal a striking enrichment of long poly dA.dT tracts in intergenic regions (IGR) compared to expected frequencies of random tracts of equivalent base composition [1–3]. Utilizing techniques such as MAINE-FAIRE (micrococcal nuclease-assisted isolation of nucleosomal elements and formaldehyde assisted isolation of regulatory elements) or chromatin immunoprecipitation, both coupled to high throughput sequencing approaches, reveal a relative depletion of nucleosomes over poly dA.dT tracts of increasing length [2, 4–6]. The shorter helical repeat distance within poly dA.dT tracts, along with a narrower and deeper minor grove with a defined spine of hydration, appear to energetically disfavour the necessary remodeling of these tracts for nucleosome binding (for review see [7]). Over recent years, high-resolution maps of nucleosome positioning in a number of eukaryotes have revealed that these poly dA.dT tracts represent a canonical feature of an intrinsic nucleosome positioning code within DNA sequences [4, 5, 8–11]. A second canonical feature, a 10 bp periodicity of AA/TT/TA dinucleotide repeats, provides an opposing role by promoting sharp bending of DNA necessary for wrapping around nucleosomes. Thus, the absence of dinucleotide repeats, together with the presence tracts of poly dA.dT, provides for an ordered, and modifiable, nucleosome landscape where nucleosome depleted regions (NDR) typically act as barriers between well-ordered arrays of nucleosomes organized over exonic sequences [5, 7, 9, 10]. Critically, these flanking 5’ and 3’ NDR, located in the immediate IGR, provide key sites for the regulation of gene expression. 5’ NFR facilitating access of specific transcription factors and the basal transcriptional apparatus to the genomic template, with the more recently described 3’ NDR offering a region for RNA polymerase II (RNAPolII) complex disassembly over transcription termination sites as well as a site for initiation of antisense transcription [4, 11].

Analysis of chromosomes 2 and 3 of *Plasmodium falciparum*, the aetiological agent of the most virulent form of human malaria, reveals higher than expected frequencies of over-long polydA.dT tracts in IGR [1, 3]. Whilst few studies have explored the function of poly dA.dT tracts in the control of gene expression in this parasite, those that have support a role for these tracts in altering absolute levels of gene expression [12, 13]. Genome-wide profiling of nucleosome positioning during the intraerythrocytic (IE) stage of development indicates variation in positioning subject to temporal developmentally-linked control [14–16]. Maximal levels of nucleosome occupancy are apparent in the final stages of IE development with minimum nucleosome occupancy levels coincident with S-phase and the highest levels of transcriptional activity [16]. *P. falciparum* is unusual in that the majority of its genome is maintained in a euchromatic state, with no evidence of the highest orders of chromatin packing as a result of the apparent absence of histone H1 (for reviews see [17, 18]). Given recent findings that suggest the spatial organization of chromatin within the nucleus affects gene expression [19], it appears likely that dramatic macro-scale rearrangement of chromatin accompanied by micro-scale nucleosome rearrangements over IGR play a major role in directing the cascade of developmentally-linked mRNA steady state levels during intraerythrocytic schizogony. Spatial variations in nucleosome occupancy in *P. falciparum* are generally apparent over key expression landmarks [16, 20]. Nucleosomes are preferentially distributed over exonic sequences whilst intergenic regions immediately flanking exonic sequences are relatively nucleosome depleted [14–16]. To date, however, no evidence of a spatial correlation between poly dA.dT tracts and the positioning of these gene-flanking NFR has been demonstrated. Moreover, whilst in other organisms the 5’ flanking NDR typically contain the transcription start site, this is unlikely in *P. falciparum*. Precise mapping of transcriptional start sites is challenging within the extreme AT nucleotide content of *P. falciparum* intergenic regions (typically exceeding 80-90%), although a recent study performed by us suggests that these are likely located some 600–1350 bp upstream of the start of the coding sequence [21–23], at least 300-400 bp beyond the mapped 5’ NDR border.

**Figure 1:**
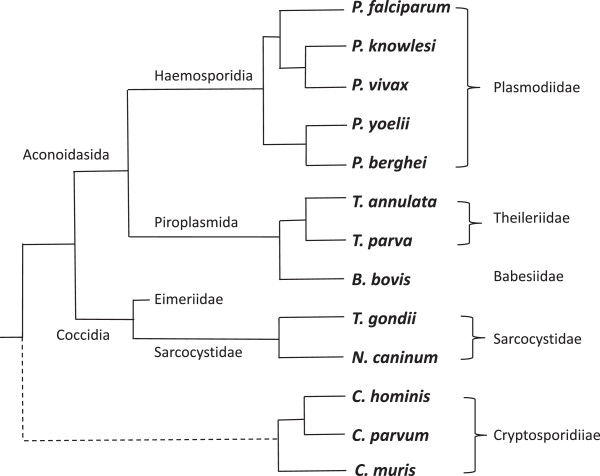
**Cladogram of Apicomplexan organisms used in this study.** Note the branches of the cladogram are incomplete and are intended only to display relative relationships between the 13 organisms investigated. The dotted branch lines for *Cryptosporidium spp.* indicate the undefined relationship of these early branching Apicomplexan parasites that lack the apicoplast organelle. Information to the left provides information regarding order/sub-order, with the families of these organisms reported to the right.

The global impact on human and animal health imposed by the protist parasites of the Apicomplexan phylum, of which *P. falciparum* is perhaps the best-known member, has driven a sustained effort to sequence their genomes over recent years. Thus, complete annotated genomes are available for: (i) the human malaria parasites *P. falciparum, P. vivax* and *P. knowlesi* as well as murine models for this disease (*P. berghei* and *P. yoelii*), (ii) the closely related hematozoans of the order Piroplasmarida *Theileria spp.* and *Babesia bovis*, aetiological agents of the bovine diseases tropical theileriosis, East Coast fever and babesiosis, (iii) three genomes for *Cryptosporidium spp*., aetiological agents of a life-threatening diarroheal disease in immunocompromised individuals and (iv) two additional coccidian parasites of the sub-order Eimeriorina, *Toxoplasma gondii* and *Neurospora caninum*, responsible for abortive diseases in sheep and cattle, respectively (see Figure 1 for cladogram of these organisms) [20, 24–33]. These resources offer an opportunity to perform a detailed comparative analysis of homopolymeric tract organisation within IGR distinct from previous comparative studies that have emphasised evolutionary diversity in the organisms investigated. In a recent report exploring the size and organisation of IGR within the Apicomplexan phylum, we demonstrated a consensus gene-spacing rule that is shared between the moderately compact genomes in this phylum despite the huge variation in the sizes of their genomes (8-63Mbp) [22]. That is, the size of IGR reflects the nature of the core transcriptional activity over the IGR; group A IGRs, containing two promoters (head-to-head flanking genes), are larger than group B IGRs, which contain one promoter and one terminator (head-to-tail flanking genes), and these in turn are larger than type C IGRs that contain two terminators (tail-to-tail flanking genes). For those parasite genomes with moderately compact gene density (*c.* 2.0-4.8kbp/ORF), we showed that irrespective of the actual mean sizes of the IGR there is a consensus 3:2:1 ratio in the median size of types A:B:C IGR.

This study was designed to address two aims. First, provide a comparative analysis of homopolymer tract frequency, size and spatial organisation in what may be considered functionally comparable regions of flanking IGR in these evolutionary-related pathogenic organisms. The second aim then correlates the spatial organisation of poly dA.dT tracts in *P. falciparum* with available nucleosome mapping data to explore a role for poly dA.dT tracts in directing nucleosome positioning over NDR associated with key expression landmarks.

## Results

### Comparative analysis of the representation of homopolymer tract frequencies in the proximal intergenic regions of Apicomplexan parasites

**Figure 2:**
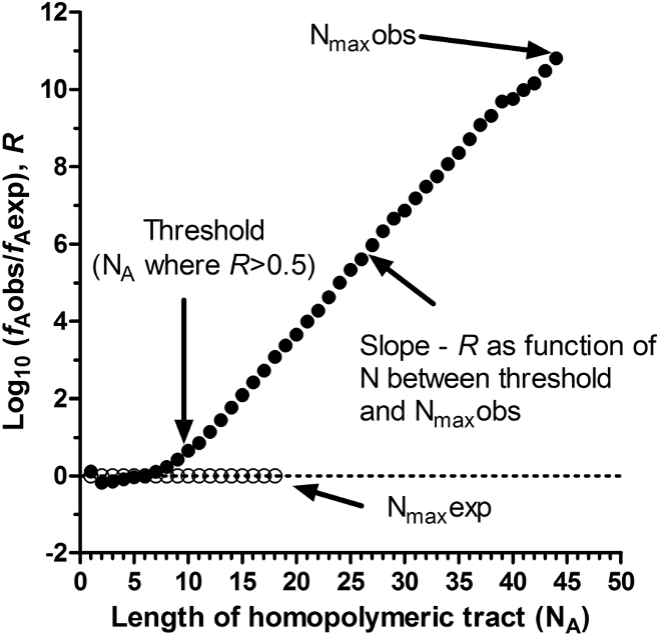
**Example output from Poly analysis of homopolymer tract frequency.** The representation, *R*, (f_A_obs/f_A_exp) of poly dA tracts is plotted as a function of their length (N_A_). Filled circles represent the data obtained from an analysis of up to 2000 bases of sense-strand sequence upstream of open reading frames in *P. falciparum*. Unfilled circles represent data obtained from randomised shuffled sequences of the same length and nucleotide content. N_max_obs is the longest length of poly dA observed (where a minimum of four tracts of this length were observed) in the upstream sequence, with N_max_exp representing the maximal length of poly dA tract expected from random base sequence of the same length and nucleotide content. The threshold is the length of poly dA tract which is observed to be over-represented (using the *R* > 0.5 criterion) in the upstream sequences. The slope of overrepresentation (slope_*R*_) is determined from data between the threshold and N_max_obs points.

To explore homopolymeric tract organisation in functionally-comparable regions of flanking IGR, the median size of type A IGR (promoter) and type C IGR (terminator) of sense-strand flanking sequences upstream and downstream, respectively, of each ORF in 13 Apicomplexan parasite species were obtained (Additional file 1: Table S1). In each case, flanking sequences were obtained up to a maximum length (correlating to the median size of the IGR as indicated in Additional file 1: Table S1) unless the adjacent ORF was encountered, in which case, only the flanking sequence up to the ORF was taken. The open source algorithm Poly (see Methods) was used to search for and provide quantitative data on the frequencies of homopolymer tracts nucleotides A, G, C and T (collectively termed i) for all lengths N [3, 34]. These data include: (1) the fraction of each nucleotide i within these sequences (Fraction_i_), (2) the frequency of each tract i of length N bases observed (*f* i_N_obs) as well as that expected (*f* i_N_exp) from randomized sequences of the same length and nucleotide content, (3) the maximal length of each homopolymer tract observed (N_max_obs, where the occurrence of N_max_obs was ≥ 4) and that expected (N_max_exp), again, from randomized sequences of the same length and nucleotide content (Figure 2 and Table 1). These data enable various aspects of the relative frequency and length of all different homopolymer tracts in these proximal flanking regions to be described. First, their representation, *R*, which provides a measure of the observed frequency of each tract length (*f* i_N_obs) normalised against that expected by random occurrence (*f* i_N_exp). Given that homopolymeric tracts in intergenic regions are highly overrepresented, this is plotted as log_10_(*f* i_N_obs/ *f* i_N_obs) vs N (Figure 2). Another measure involving *R* is the threshold of overrepresentation, a particular value of *R* and tract length N where the relationship *f* i_N_obs > *f* i_N_exp achieves significance. Here the threshold is set at *R* ≥ 0.5, i.e. the observed frequency is 10^0.5^ (3.16-fold) higher than that expected by chance from randomized sequences. The second aspect, proportion (*P*), provides a description of the relative length of the longest observed tract i compared to that expected from randomized sequences, and is derived from N_max_obs/N_max_exp (Table 1).

**Table 1:** **Summary of POLY analyses of upstream and downstream gene-flanking sequences in Apicomplexan parasites**

Organism	Fraction _i_				Maximum length observed N _max_obs				Maximum length expected M _max_exp				Proportionment ( ***P***)				Threshold (R > 0.5)				Slope _R_^1^			
	A	C	G	T	A	C	G	T	A	C	G	T	A	C	G	T	A	C	G	T	A	C	G	T
***Upstream***																								
*P.falciparum*	0.43	0.06	0.07	0.44	444	9	9	43	18	5	5	19	2.44	1.80	1.80	2.26	10	3	4	10	0.31	0.72	0.74	0.29
*P.knowlesi*	0.30	0.19	0.18	0.31	46	17	17	47	13	9	9	13	3.54	1.89	1.89	3.62	7	5	5	7	0.46	0.49	0.48	0.44
*P.vivax*	0.27	0.22	0.21	0.29	35	17	16	36	12	10	10	12	2.92	1.70	1.60	3.00	6	5	5	6	0.47	0.36	0.35	0.45
*P.yoelii*	0.42	0.10	0.12	0.36	35	10	7	32	18	7	7	16	1.94	1.43	1.00	2.00	10	4	5	8	0.32	0.57	0.40	0.32
*P.berghei*	0.39	0.10	0.10	0.40	23	10	7	24	16	6	6	16	1.44	1.67	1.17	1.50	8	4	4	8	0.25	0.57	0.48	0.23
*C.hominis*	0.36	0.14	0.15	0.35	18	9	9	18	14	7	7	13	1.29	1.29	1.29	1.38	9	5	6	8	0.24	0.46	0.39	0.26
*C.parvum*	0.37	0.13	0.14	0.36	22	9	10	na^2^	14	7	7	na	1.57	1.29	1.43	na	9	5	5	na	0.26	0.45	0.47	na
*C.muris*	0.36	0.13	0.14	0.37	20	10	11	21	14	7	7	14	1.43	1.43	1.57	1.50	10	6	6	10	0.30	0.60	0.67	0.30
*T.gondii*	0.23	0.26	0.26	0.25	13	17	17	na	11	12	12	na	1.18	1.42	1.42	na	6	10	10	na	0.08	0.29	0.29	na
*N.caninum*	0.22	0.27	0.26	0.25	11	20	21	11	10	12	12	11	1.10	1.67	1.75	1.00	5	10	10	6	0.14	0.47	0.51	0.10
*B.bovis*	0.31	0.19	0.20	0.30	9	7	7	10	12	8	8	12	0.75	0.88	0.88	0.83	na	na	na	na	na	na	na	na
*T.annulata*	0.37	0.13	0.13	0.37	11	6	6	11	14	6	7	14	0.79	1.00	0.86	0.79	na	4	7	na	na	na	na	na
*T.parva*	0.36	0.14	0.15	0.35	9	6	6	9	14	7	7	13	0.64	0.86	0.86	0.69	na	5	na	na	na	na	na	na
***Downstream***																								
*P.falciparum*	0.41	0.07	0.07	0.44	43	7	8	45	17	5	5	18	2.53	1.40	1.60	2.50	10	4	4	10	0.32	0.59	0.63	0.29
*P.knowlesi*	0.29	0.19	0.19	0.33	42	16	16	44	12	9	8	13	3.50	1.78	2.00	3.38	6	5	5	7	0.46	0.49	0.48	0.44
*P.vivax*	0.26	0.23	0.22	0.29	32	15	16	32	11	10	10	11	2.91	1.50	1.60	2.91	6	5	5	6	0.50	0.39	0.40	0.45
*P.yoelii*	0.38	0.12	0.11	0.39	33	9	8	31	15	6	6	16	2.20	1.50	1.33	1.94	8	4	4	8	0.33	0.45	0.34	0.31
*P.berghei*	0.38	0.11	0.10	0.41	24	7	9	22	14	6	6	16	1.71	1.17	1.50	1.38	8	4	4	8	0.25	0.34	0.50	0.22
*C.hominis*	0.35	0.14	0.13	0.38	13	7	7	16	12	6	6	13	1.08	1.17	1.17	1.23	9	6	6	9	0.20	0.42	0.49	0.20
*C.parvum*	0.35	0.13	0.13	0.39	15	7	7	19	12	6	6	14	1.25	1.17	1.17	na	9	6	5	9	0.21	0.37	0.36	0.21
*C.muris*	0.37	0.12	0.12	0.39	17	6	7	18	13	6	6	14	1.31	1.00	1.17	1.29	10	5	6	11	0.30	0.65	0.50	0.26
*T.gondii*	0.25	0.25	0.25	0.25	11	15	17	11	11	11	11	12	1.00	1.36	1.55	na	7	10	10	7	0.06	0.43	0.47	0.01
*N.caninum*	0.24	0.25	0.27	0.24	11	21	24	11	11	11	12	11	1.00	1.91	2.00	1.00	6	10	10	6	0.11	0.54	0.51	0.11
*B.bovis*	0.30	0.19	0.19	0.31	9	7	7	8	11	8	8	11	0.82	0.88	0.88	0.73	na	na	na	na	na	na	na	na
*T.annulata*	0.36	0.12	0.13	0.38	10	7	6	9	13	6	6	13	0.77	1.17	1.00	0.69	na	5	na	na	na	na	na	na
*T.parva*	0.35	0.13	0.14	0.38	9	7	6	9	12	6	6	13	0.75	1.17	1.00	0.69	na	5	na	na	na	na	na	na

^1^slope of *R* between threshold of overrepresentation and N_max_obs. ^2^na, Not available.

Plotting *R* as a function of tract length N reveals three distinct organisations of homopolymer tracts in the proximal intergenic flanking regions of these Apicomplexans, with related organisms generally sharing the same organisation (Additional file 1: Figure S1 and S2). The first, shared by the *Plasmodium spp*. and *Cryptosporidium spp*., shows the typical eukaryotic organisation of overrepresentation of short poly dG.dC tracts and long poly dA.dT tracts [3]. This pattern is reversed in the second organisation shared by the coccidian parasites *N. caninum* and *T. gondii*, where instead short poly dA.dT tracts and long poly dG.dC tracts are overrepresented. The final organisation is shared by the piroplasmida *Theileria spp*. and *B. bovis*, where there is no evidence of overrepresentation of any homopolymer tracts, with the longest poly dA.dT tracts in *Theileria spp*. actually reaching the threshold for underrepresentation (*R* ≥ −0.5). Of note, however, is that despite the dissimilarities in the length of proximal upstream and downstream flanking sequence secured from each organism, there appears to be no difference between them in terms of the maximum level of *R*, threshold of overrepresentation or N_max_obs (Table 1).

**Figure 3:**
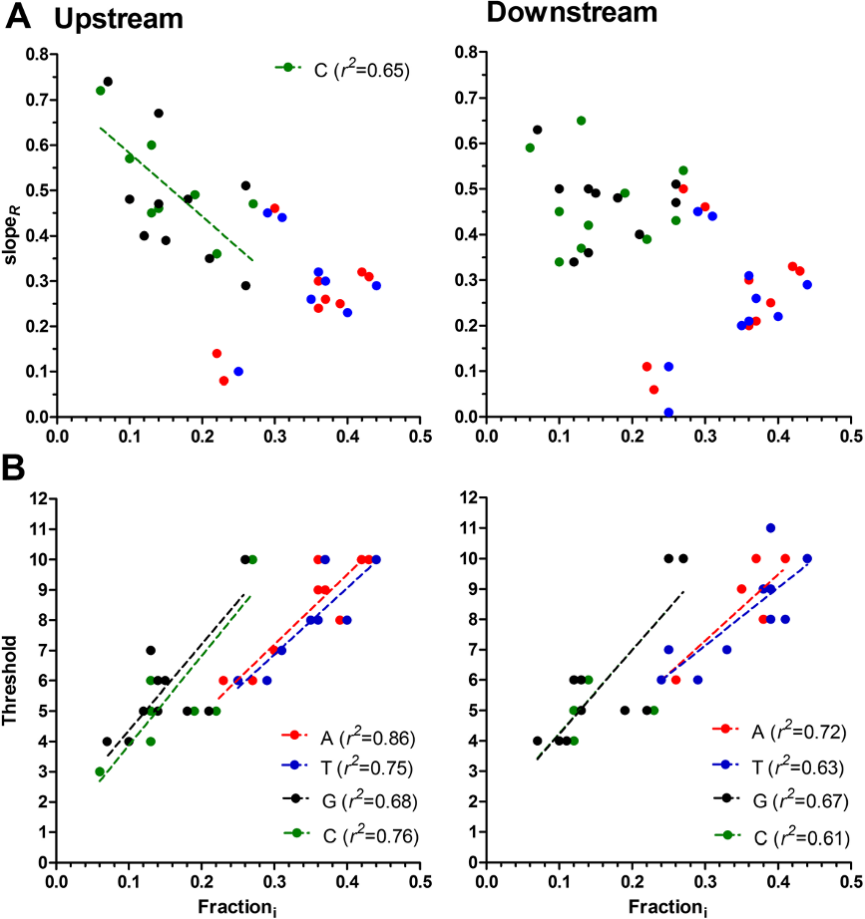
**Comparative analysis of overrepresentation of homopolymeric tracts as a function of nucleotide content. (A)** The slope of overrepresentation (slope_*R*_) of each homopolymer tract (see key in B) plotted as a function of the fraction of each nucleotide content (Fraction_i_) in upstream and downstream proximal flanking regions. Only poly dC tracts in upstream proximal flanking regions show a significant correlation as indicated. **(B)** The threshold of homopolymer tract_i_ plotted as a function of Fraction_i_ in upstream and downstream proximal flanking regions. All linear regression analyses (dotted lines) were significant, with the coefficient of correlation (*r* ^*2*^) reported in the key.

To compare the relative levels of overrepresentation of the different homopolymeric tracts between these species against the backdrop of their diverse nucleotide content, the slope of *R* (slope_*R*_) was determined (Figure 2 and Table 1). In general, as Fraction_i_ increases, slope_*R*_ would decrease as the denominator *f* i_N_exp increases. Comparison of slope_*R*_ for all homopolymeric tract types in upstream and downstream intergenic flanking sequences (Figure 3A) reveals that this general trend is present (*r*^2^ = 0.33, *p* < 0.001, slope = −0.80). However, reanalysis of this trend for each homopolymer type reveals no significant correlation, excepting polydC in the upstream region (Figure 3A). Thus, the three distinct organisations of overrepresented homopolymeric tracts is not a reflection of any difference in nucleotide content between these organisms, but instead appears to reflect an inherent difference in the way homopolymer tracts are organised within the different families of the Apicomplexa phylum.

Previous analysis of the threshold length for overrepresentation of homopolymeric tracts in eukaryotic intergenic sequences reveals a positive correlation with Fraction_i_ [1, 3]. That is, as Fraction_i_ increases the length of N_i_ necessary to meet the threshold of overrepresentation also increases. This is also apparent here (Figure 3B, all regression lines p < 0.01) in the more defined proximal intergenic sequences investigated in these Apicomplexan organisms, irrespective of any differences in how the homopolymer tracts are overrepresented (nb. Piroplasmida are not included in this analysis as homopolymeric tracts are not overrepresented). Regression analysis reveals no significant difference between the slopes of poly dA.dT or poly dG.dC when compared between upstream and downstream proximal intergenic sequences (all *p* > 0.15). The slopes estimated for poly dA.dT and poly dG.dC tracts are, however, significantly different from each other (*p* < 0.0001), which would suggest that at an equivalent Fraction_i_, poly dA.dT are more likely overrepresented at a shorter tract threshold than are poly dG.dC tracts.

### Overproportionment of poly dA.dT tracts correlates with the size of flanking intergenic regions in more compact genomes

**Figure 4:**
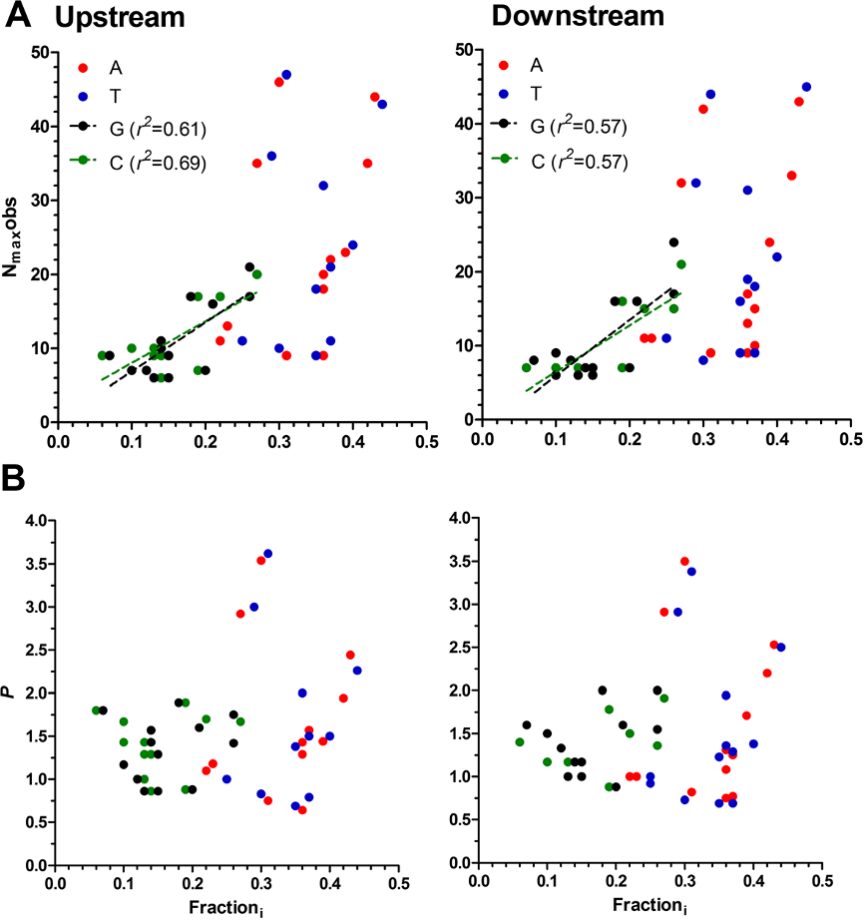
**Comparative analysis of overproportionment of homopolymeric tracts as a function of nucleotide content.** Plots of **(A)** N_max_obs and **(B)** proportionment (*P*) of each homopolymer tract (see key in A) as a function of Fraction_i_ in upstream and downstream proximal flanking regions. Only the N_max_obs of poly dG.dC tracts show a significant linear correlation. This is represented using dotted lines with the coefficient of correlation (*r* ^*2*^) reported in the key.

**Figure 5:**
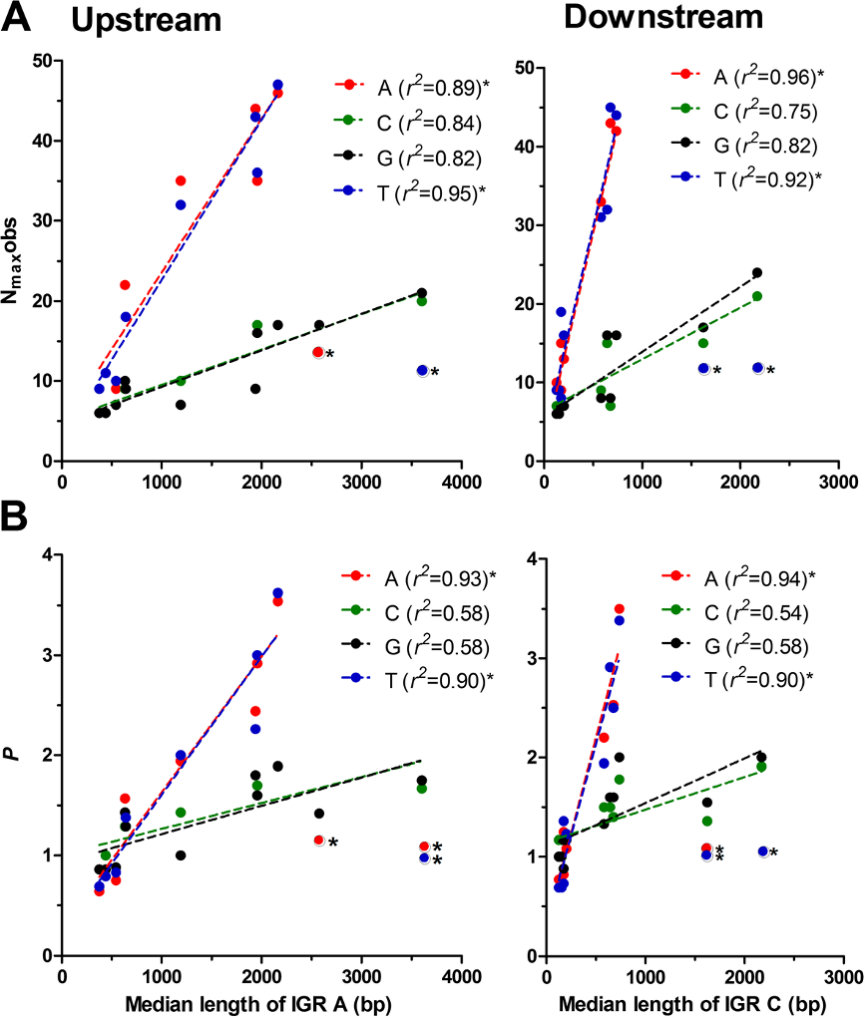
**Comparative analysis of overproportionment of homopolymeric tracts as a function of size of intergenic region (IGR).** Plots of **(A)** N_max_obs and **(B)** proportionment (*P*) of each homopolymer tract (see keys) as a function of the median length of upstream (type A IGR) and downstream (type C IGR) proximal flanking regions. All significant linear correlations are represented using dotted lines with the coefficient of correlation (*r* ^*2*^) reported in the respective key for that panel. Note, poly dA.dT points indicated with asterisks (*T. gondii* and *N. caninum*) were omitted from the linear regression analysis; the *r* ^*2*^ reported also omits these values).

The value of N_max_obs for any tract type would be expected to increase as both Fraction_i_ and the length of the sequences being investigated increase. Using the denominator N_max_exp to define proportion, *P*, (N_max_obs/N_max_exp) facilitates a comparative analysis of maximum homopolymer tract length, independent of nucleotide composition and tract length. Plotting N_max_obs of poly dG.dC tracts as a function of either Fraction_i_ or the median size of intergenic sequence (nb. as this increases, longer sequence lengths are obtained for analysis) reveals the anticipated positive correlation in both upstream and downstream proximal flanking sequence populations (Figures 4A and 5A). Normalising N_max_obs to determine *P*, reveals the expected levels of overproportionment of poly dG.dC tracts (*P* between 0.8 to 1.9) in intergenic regions (Figure 4B). *P* for poly dG.dC tracts does not correlate with nucleotide composition, and whilst there is a significant coefficient of correlation between *P* and length of intergenic regions, the negligible elevation of the slope suggests *P* is largely independent of length of the upstream and downstream proximal intergenic sequences investigated here (Figure 5B).

The N_max_obs of poly dA.dT tracts as a function of Fraction_i_ shows no significant correlation (Figure 4A): i.e. the maximum length of poly dA.dT tracts is not simply dependent upon how AT rich a genome is. The highly overproportioned (*P* between 1.44 and 3.62) poly dA.dT tracts found in *Plasmodium spp*., irrespective of the AT-content in the proximal flanking regions, clearly biases this analysis (Figure 4B). Plotting N_max_obs as a function of the median intergenic distance also initially reveals no significant correlation, primarily due to the absence of overproportioned poly dA.dT tracts in the large intergenic distances found in *T. gondii* and *N. caninum*. Here, the proportionally longer type C intergenic regions (two terminators) in *T. gondii* and *N. caninum* collapses the 3:2:1 space apportionment ratio typical of the other Apicomplexan parasites investigated (Additional file 1: Table S1), all of which share a more compact genome density (2.0-4.6 kb/gene) than that of these coccidian parasites (8.6-9.1 kb/gene) [22]. Excluding *T. gondii* and *N. caninum*, however, reveals a strong positive correlation between both N_max_obs and *P* of poly dA.dT tracts as intergenic distances increase in length (Figure 5, fit lines shown). Thus, the overproportionment of the long poly dA.dT tracts in *Plasmodium spp*. is not a reflection of any bias in their AT content, but is perhaps instead a reflection of the longer lengths of their intergenic sequences. This observation goes some way to explain why the N_max_obs of poly dA.dT tracts in *P. falciparum* and *P. knowlesi* are similar; despite *P. knowlesi* proximal flanking sequences having a much lower AT-content than those of *P. falciparum*, as the median intergenic distances in *P. knowlesi* are longer.

### Homopolymer tracts are overrepresented and overproportioned in the open reading frames of *Plasmodium spp*

Previous analysis of homopolymer tract organisation in *P. falciparum* ORF reports the overrepresentation and overproportionment of all homopolymer tract types, but poly dA.dT tracts in particular [1, 3]. To extend this analysis, ORF were obtained for all (except *N. caninum*) of the Apicomplexan parasites investigated here and the program Poly was used to search for and provide quantitative data for the frequency of all homopolymer tract types (Additional file 1: Table S1). For comparison, a Poly analysis of human and mouse ORF were included, as these organisms represent the general eukaryotic pattern of absence of any homopolymeric tracts in these sequences [1, 3].

**Figure 6:**
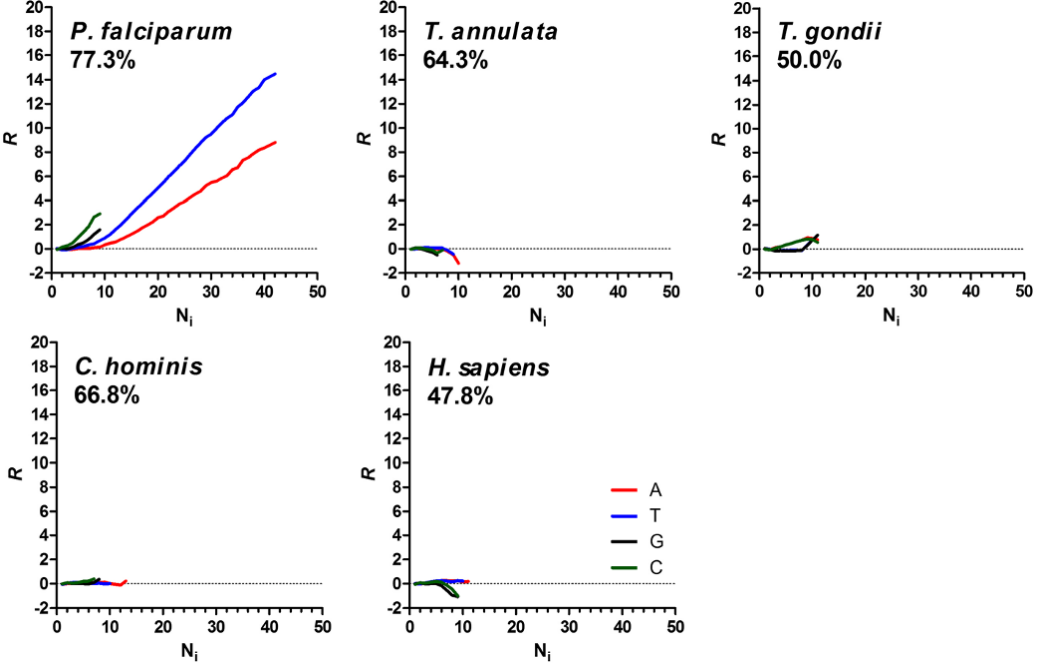
**Representation of homopolymeric tracts over coding sequences.** These graphs plot *R* for homopolymer tracts (see key, lower right) as a function of their length (N_i_). The species as well as the average%AT content of the coding sequences analysed is reported on each graph.

Our analysis reveals that, like human and mouse ORF, the ORF of cryptosporidium, piroplasmida and coccidian parasites show no evidence of overrepresented or overproportioned homopolymeric tracts (Figure 6 and Additional file 1: Figure S3). In fact, these tracts instead tend to be underrepresented and underproportioned, particularly at longer tract-length, reflecting the impact of codon usage in these coding sequences. All the *Plasmodium spp*., however, show extensive overrepresentation and overproportionment of all homopolymeric tracts in ORF sequences, and again of poly dA.dT in particular (Additional file 1: Figure S3). As expected, overrepresentation and overproportionment of tracts is greater in human malarial parasites (eg. poly dA.dT N_max_obs range between 29–42 with *P* between 2.1-3.82) than murine malarial parasites (eg. poly dA.dT N_max_obs range between 17–29 with *P* between 1.31-1.69) reflecting the larger average size of ORF in human compared to murine malarial parasites.

### Spatial analysis of poly dA.dT tracts in proximal flanking intergenic sequences

Given the distinct overrepresentation and overproportionment of poly dA.dT tracts in the proximal intergenic sequences of three of the six *Plasmodium spp*. that are the aetiological agents of human malaria, these organisms were selected for an initial analysis of the spatial organisation of these homopolymer tracts using the frequency counting program motif.freq.pl (see Methods). Upstream (2000 bases) and downstream (700 bases) sense-strand sequences for all genes were distributed into sequential 50 base length bins for a count of the occurrence of non-overlapping poly dA.dT tracts of N = 5, 10, 15 and 20 base length. Given that the total number of nucleotides in each bin will decrease as the distance of the bin from the ORF increases (nb. the probability of encountering an adjacent ORF increases as you move away from the ORF of interest, and only intergenic sequence are captured for analysis here), the number of tracts observed is normalised by the total number of nucleotides in the bin (representing the bin frequency counting output of motif.freq.pl) to provide a useful comparative measure of the observed frequency (F_obs_) of these non-overlapping tracts.

**Figure 7:**
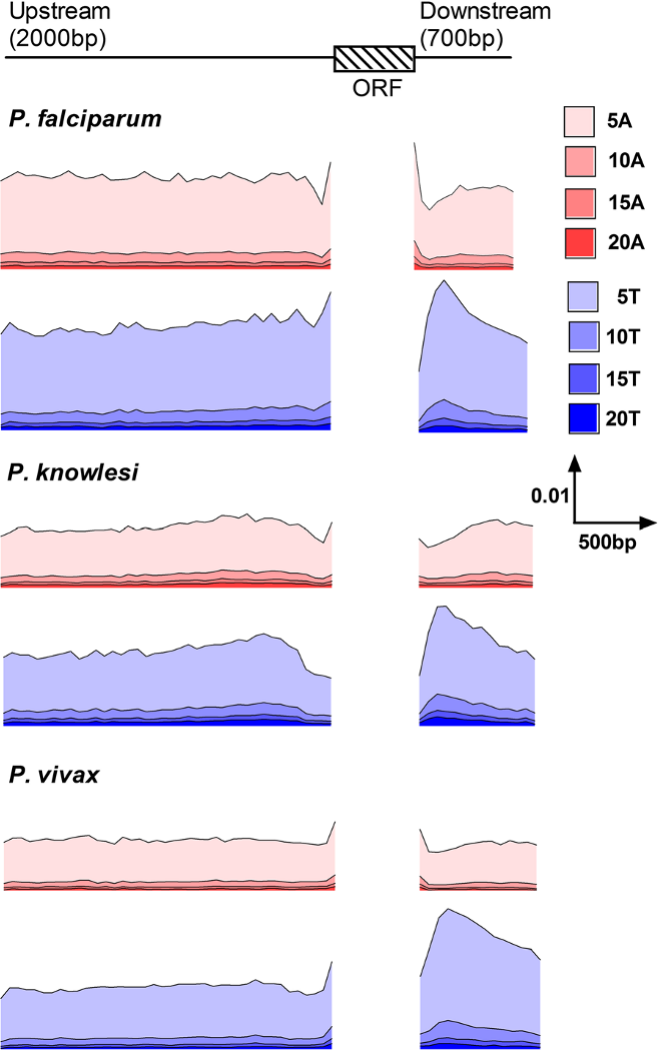
**Spatial distribution of poly dA.dT tracts in proximal flanking intergenic regions of three** ***Plasmodium spp.*** The schematics represent the distribution (bin size of 50 bases) of homopolymer tracts of N = 5, 10, 15 and 20 bases (see key for base and size of tract) in the sense-strand of proximal upstream and downstream intergenic sequences. Distance from the open reading frame (ORF) is represented on the X-axis. The frequency of each tract (F_obs_) is represented on the Y-axis. The scale for distance and F_obs_ is represented in the key.

**Figure 8:**
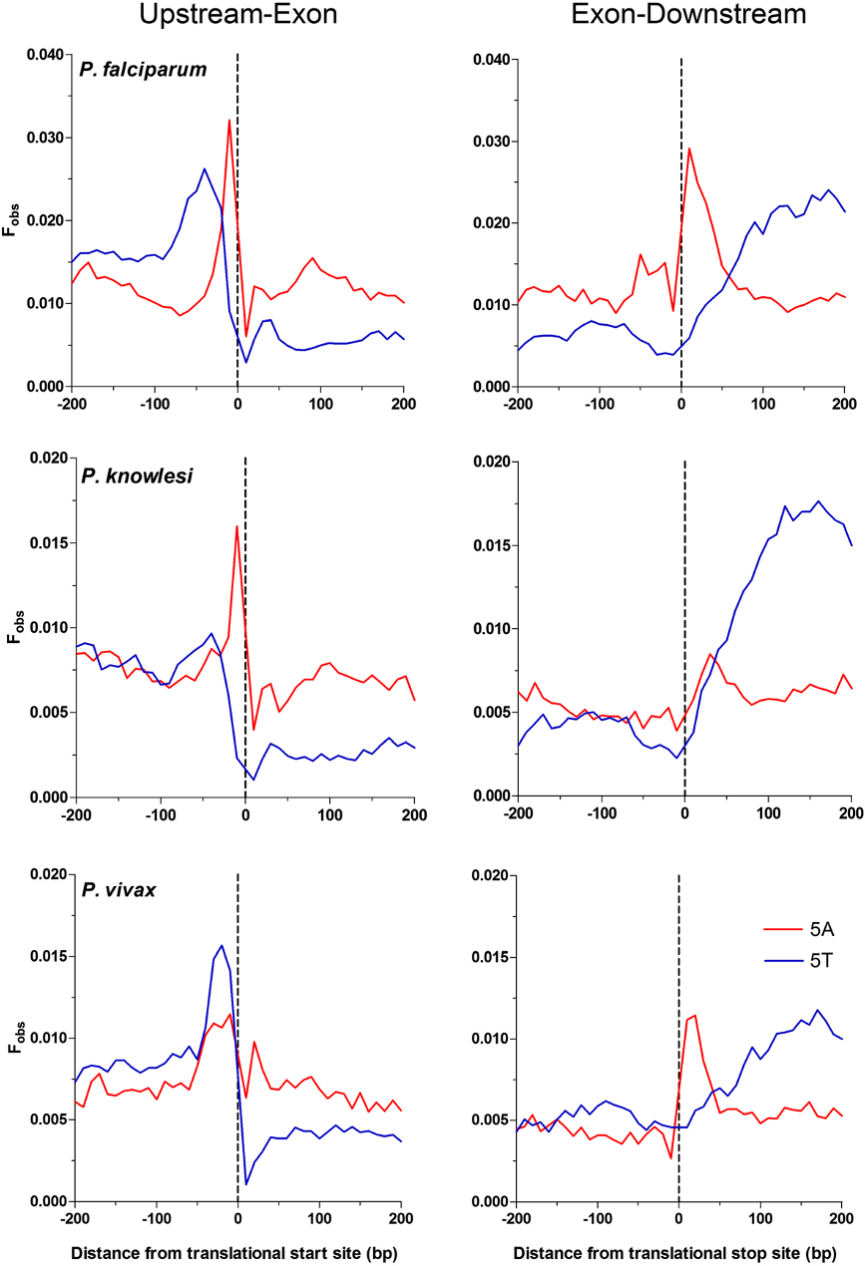
**Spatial distribution of poly dA.dT tracts immediately flanking the ORF from three** ***Plasmodium spp.*** Plots of the spatial distribution (bin size of 10 bases, X-axis) of frequency (F_obs_) of poly dA (red line) and poly dT (blue line) tracts of 5 base length in the 200 bases of sense strand flanking either side of the translational start (upstream-exon) and stop (exon-downstream) sites.

Comparison of the F_obs_ of the different lengths of poly dA.dT tracts in both upstream and downstream proximal flanking regions shows the expected decrease as (i) the Fraction_i_ of nucleotides A and T decreases from *P. falciparum* to *P. vivax* and (ii) as the length of tract investigated increases (Figure 7). Irrespective of the *Plasmodium spp*. investigated, the spatial distribution in F_obs_ for all tract lengths only varies in the flanking regions immediately adjacent to the ORF (i.e. within 200 bp). A higher-resoultion determination of F_obs_ was therefore carried out on the 200 bases that flank either side the start and end of all ORF in these *Plasmodium spp.* to refine this observation (Figure 8, tract size N = 5, 10 base bins). This analysis reveals a clear pattern of spatial arrangement of poly dA.dT tracts on the sense strands immediately prior to and following the ORF. Poly dA tracts are more likely observed within 10-20 bp either side of the ORF, with peaks of F_obs_ for poly dT tracts more distally located, approximately 50 bp upstream of the start codon and 50-200 bp downstream of the stop codon. Accounting for the differences in AT content between these three *Plasmodium spp*., an “expected” frequency of occurrence of N = 5 poly dA.dT tracts can be estimated from a repeated 10× random shuffle of the sequences using the program shuffle.pl (see Methods), where the 10X shuffled frequencies are averaged to produce F_10xshuffle_, ensuring that the Fraction_i_ of all nucleotides is maintained during shuffling but not the integrity of homopolymer tracts. Plotting the normalized frequency ratio, F_obs_/F_10xshuffle_, accounts for base composition bias and the plots show that as the Fraction_i_ of A and T nucleotides decreases from *P. falciparum* to *P. vivax*, the ratio for these peaks actually becomes greater (Additional file 1: Figure S4). That is, these spatial features of poly dA.dT tract organisation are inherent structural features and occur irrespective of AT-bias.

**Figure 9:**
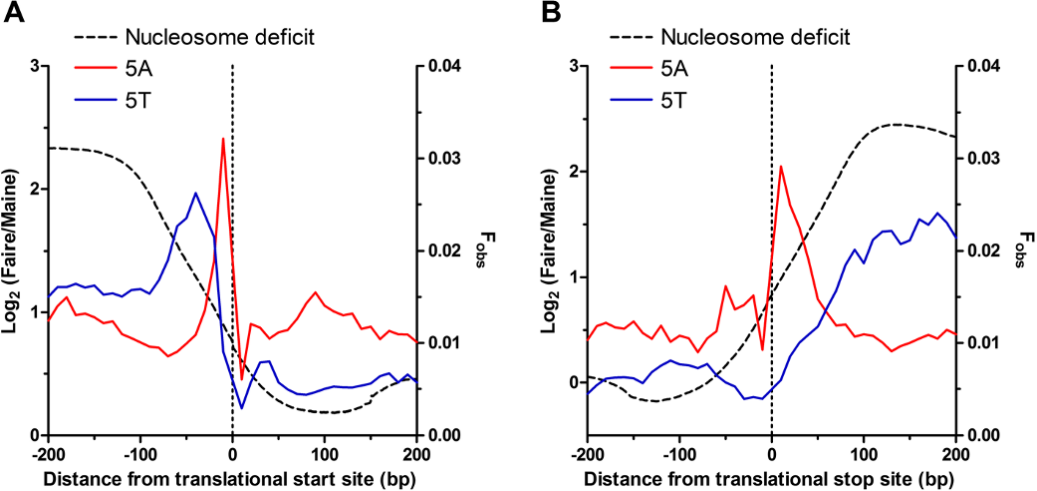
**Spatial distribution of nucleosome occupancy and poly dA.dT tracts over sequences immediately flanking** ***P. falciparum*** **ORF.** Plots of the spatial distribution of observed frequency (F_obs_, right Y-axis) of N = 5 (10 base bins) length poly dA (red line) and poly dT (blue line) tracts over 400 bp centered over translational start (**A**, upstream-exon) and stop (**B**, exon-downstream) sites. Nucleosome occupancy (black dotted line) is reported as a log_2_ ratio of FAIRE to MAINE sequence reads (left Y-axis) where an increase in the ratio indicates regions relatively deficient in nucleosome occupancy.

Genome-wide maps of nucleosomal occupancy are available for *P. falciparum* [14, 15]. To compare nucleosome occupancy with these spatially organised poly dA.dT tracts around the translational start and stop sites, a log_2_ ratio of existing next generation high throughput sequence reads from formaldehyde-assisted isolation of regulatory elements, FAIRE, (representing nucleosome free DNA) and micrococcal nuclease-assisted isolation of nucleosomal elements, MAINE, (representing nucleosome bound DNA) are provided to indicate relative nucleosome deficiency over this region [20]. As expected from similar analyses in other eukaryotes, the position of the proximal flanking poly dA.dT tracts correlate with the borders of the NDR located both upstream and downstream of the ORF (Figure 9).

Interestingly, this same relative spatial organisation of poly dA.dT tracts immediately adjacent to the ORF is found in the other Apicomplexan species (Additional file 1: Figure S5) investigated here. Thus, whilst baseline F_obs_ values may vary, reflecting known relative differences in overrepresentation of poly dA.dT tracts, there appears to be an established spatial organisation of poly dA.dT tracts immediately adjacent the start and end of an ORF in Apicomplexan parasites.

### Spatial analysis of poly dA.dT tracts over the core promoter in *P. falciparum*

**Figure 10:**
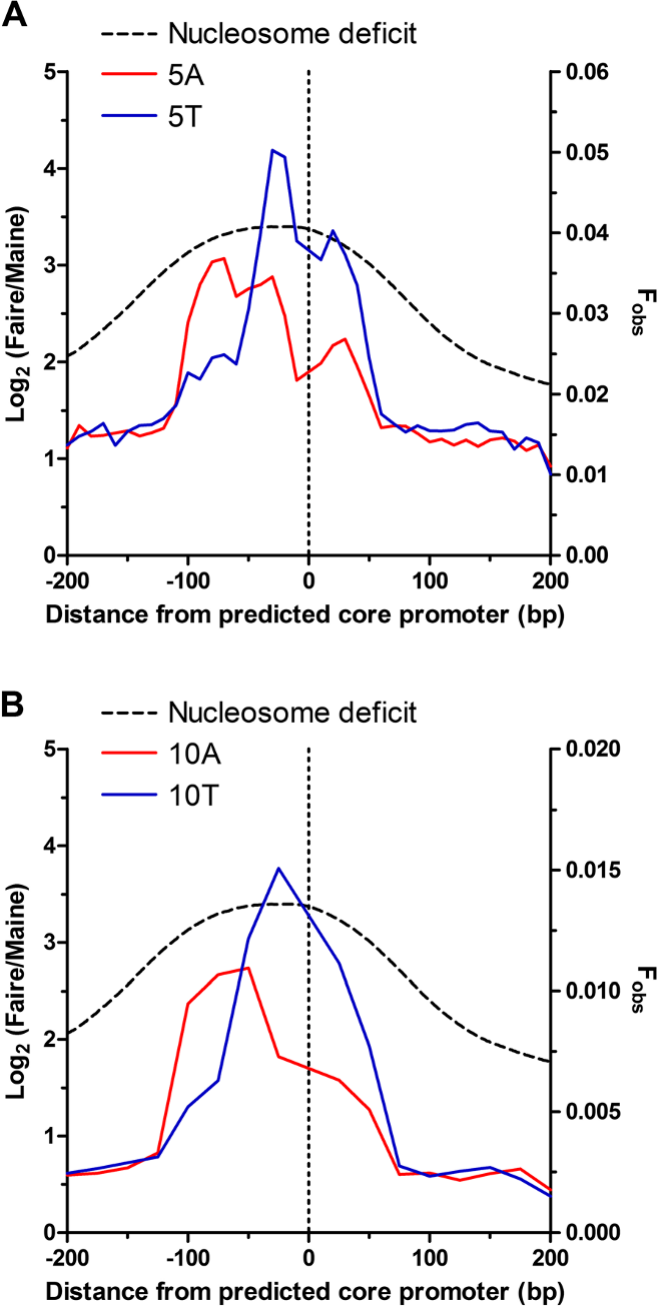
**Spatial distribution of nucleosome occupancy and poly dA.dT tracts over predicted core promoters in** ***P. falciparum.*** Plots of the spatial distribution of observed frequency (F_obs_, right Y-axis) of N = 5 (**A**, 10 base bins) and N = 10 (**B**, 25 base bins) length poly dA (red line) and poly dT (blue line) tracts from a 400 bp region centred over highly predicted core promoters. Nucleosome occupancy (black dotted line) is reported as a log_2_ ratio of FAIRE to MAINE sequence reads (left Y-axis) over the same region where an increase in the ratio indicates regions relatively deficient in nucleosome occupancy.

In the absence of unambiguously mapped transcription start sites for *P. falciparum*, available *in silico* predictions of core promoters were used to explore the spatial arrangement of poly dA.dT at this additional regulatory landmark [35]. Previous nucleosome mapping has revealed a relative deficit of nucleosome assembly centred 50 bp upstream of the most highly predicted core promoters [20]. Taking the same 3477 most confidently predicted (EGASP = 1) core promoters used to map this nucleosome depleted region, 400 bp of sequence centred on the highest scoring position for the transcription start site were secured here for an analysis of the spatial distribution of poly dA.dT tracts. Using N = 5 and N = 10-mers (10 base and 25 base bins, respectively) the F_obs_ for these poly dA.dT tracts were plotted against the distance from the predicted core promoter (Figure 10). For comparison, a log_2_ ratio of FAIRE/MAINE reads was overlaid to indicate the relative nucleosome deficiency over this same region. This analysis reveals a peak of poly dT located on the sense–strand some 10-20 bp upstream of the highest scoring position for the transcription start site, coincident with the peak of relative nucleosome deficiency. A second, but less abundant, peak of poly dA is located on the same strand some 30-50 bp further upstream of the poly dT peak. Extending this analysis to core promoters with more moderate thresholds of confidence (EGASP of 0.4-0.9) retains the same relative spatial organisation of poly dA.dT peaks to the core promoter and position of the nucleosome free region, albeit with lower F_obs_ (Additional file 1: Figure S6).

## Discussion

Simple sequence repeats, such as homopolymeric tracts, are important molecular tools in the investigation of genetic disease and evolution. As such, significant efforts have been invested in understanding the forces that shape their frequency, length, spatial distribution and function [1, 3, 35–37]. It is apparent that there is a dynamic balance between stochastic seeding and expansion of homopolymer tracts, their mutation, and non-stochastic selective pressures (e.g. intrinsic DNA factors such as triplet code, nucleosome positioning information and initiation of transcription), that together direct their evolution and persistence. Two leading theories for the seeding and expansion of homopolymer tracts invoke either slipped-strand replication errors or the action of transposable elements such as retrotransposons [38–41]. The evidence for slipped-strand replication errors directing tract expansion is initially conceptually compelling. In general, representation of homopolymeric tracts increases logarithmically when tract lengths are equal to, or exceed, approximately 7 bases – this figure apparently representing a minimal thermodynamic threshold for slipped-strand errors during replication [1]. This theory, however, was challenged in a study of homopolymeric tract frequencies in 27 organisms which showed that there is no single threshold tract-length for homopolymer overrepresentation [3]. Instead, this work demonstrated that the threshold tract-length for overrepresentation is dependent upon the base composition (Fraction_i_). Thus, for polydA.dT tracts, as Fraction_A,T_ increases, the threshold tract length for overrepresentation of these tracts similarly increases. This study of homopolymer tract frequency in Apicomplexan protozoa agrees with this observation, although our study shares with the Zhou *et al.* study [3] a similar limitation in range of average genomic AT content of organisms (between 50-85%). Whether this same correlation between threshold tract-length for polydA.dT overrepresentation and Fraction_A,T_ exists in more GC rich organisms needs to be established to explore the universality of this observation and its implication for homopolymer tract-length expansion.

The second theory hypothesises that polydA.dT tracts are seeded from polyadenylated transcripts of retrotransposons. This study of Apicomplexan protozoa presents a challenge, although perhaps not insurmountable, to this hypothesis. Whilst transposable elements are ubiquitously found in the genomes of metazoan eukaryotes, and even within lineages of protozoa closely related to Apicomplexans, there is a lack of overwhelming evidence for such transposable elements in the extant genomes of the Apicomplexan organisms investigated here [42–45]. Apicomplexans share the features of relatively small and moderately compact genomes, and are characterised by gene loss and expansion of species-specific gene families – features attributed to adaptation to a parasitic life cycle and virulence within the host. Critically, whilst there is extensive synteny within families of these parasites (e.g. comparing *P. falciparum* with *P. knowlesi*), there is minimal synteny between more distantly related members of the Apicomplexan lineage (e.g. comparing *Plasmodium* with *Cryptosporidium*) [42]. This evidence of dramatic genomic rearrangements within ancestral lineages, much more so than in other eukaryotic lineages of similar evolutionary age, coupled with evidence of widespread intron loss during ancestral Apicomplexan genome evolution, have led to the suggestion that retrotransposons were present in Apicomplexan ancestral lineages and may therefore represent the original source of polydA.dT tracts in modern genomes. That said, *Plasmodium spp*. and *Theileria spp*. share a recent common ancestor, and show diametrically opposed patterns of homopolymeric tract arrangement in their modern genomes. Thus, any model based on the action of retrotransposons seeding homopolymer tracts within ancestral Apicomplexan lineages would also have to account for the apparent absence of overrepresented homopolymeric tracts in modern *Theileria spp*. genomes.

Previous comparative studies of homopolymer tract frequency and/or positioning have emphasized phylogenetic diversity in the organisms studied, quite distinct to this study limited to a single phylum [1, 3]. However, this study reports features of homopolymer tract frequency, length and distribution common to these previous comparative analyses. Specifically, the correlation between base composition and length of sequence investigated with homopolymer tract representation, thresholds of overrepresentation and degree of overproportionment. Critically, however, analysis of representation and proportion of polydA.dT and polydG.dC tracts within the IGR reveals intriguing, and novel, lineage-specific patterns within Apicomplexans. Whilst members of the *Plasmodium* and *Cryptosporidium* families show a greater level of overrepresentation and overproportionment in polyd.A.dT tracts compared to polydG.dC tracts, a pattern generally shared with other eukaryotes, we find a reversal of this pattern in the coccidians and complete absence in the piroplasmida. The apparent deficit of homopolymeric tracts in the piroplasmida cannot be simply accounted for by the relatively short length of the IGR investigated in these organisms (150-550 bp), as short IGR (200-650 bp) are also analysed here in *Cryptosporidium spp*. [22]. Further, whilst the coccidians have the most GC-rich genomes of the Apicomplexans investigated here, and may therefore be expected to have longer polydG.dC tracts, both humans and mice share a similar base composition and clearly show higher levels of overrepresented and overproportioned polydA.dT tracts in their IGR [3]. Resolution of the impact of these distinct patterns of tract organisation will require nucleosome-positioning data currently not available for these organisms. Of note, however, is that whilst these organisms show distinct patterns of tract organisation in their IGR, the preferential spatial arrangement of polydA.dT tracts immediately flanking ORF is conserved. This organisation presumably confers a comparable role in nucleosome barrier placement at these sites, and suggests that canonical features of an intrinsic nucleosome positioning code are similar in all Apicomplexans.

In many eukaryotes, the 5’ NDR contains the transcription start site. Whilst accurate transcription start site mapping data are not currently available for *P. falciparum*, a similar placement of the transcription start site in the 5’ NDR is unlikely. *P. falciparum* transcripts contain significant untranslated regions (*c.* 800*–*1800 bases), with recent modelling performed by us indicating that some 75-80% of this is accounted for by the 5’UTR – suggesting that transcription start sites typically lie some 600 to 1350 bp upstream of the translational start site, much further than the 200-300 bp of the 5’ NDR [22]. In the absence of mapped transcriptional start sites, we instead correlated nucleosome positional mapping data and the spatial arrangement of polydA.dT with bioinformatic predictions of core promoters [20, 46]. Some caution needs to be applied to the interpretation of these data, as structural effects of homopolymer tracts on DNA bending are incorporated into the bioinformatic algorithm used to predict core promoters [46]. However, it is noteworthy that the centre of the NDR located over the most confidently predicted core promoters correlates with a peak of polydT on the sense strand, the 3’ end of which lies immediately upstream of the likely transcription start site. This arrangement is very similar to that recently described in the similarly AT rich *Dictyostelium discodieum* [47]. Here, the authors speculate that the RNA polymerase II preinitiation complex exploits the relative instability of the polyrU.dT RNA:DNA hybrid in abortive transcription, a mechanism key for directing a programme of coordinated temporal gene expression. It is therefore noteworthy that a not dissimilar programme of temporal global upregulation of RNAPolII pre-initiation complex (PIC) processivity is found in *P. falciparum* [48]. During intraerythrocytic schizogony, RNAPolII PIC is constitutively associated with promoter regions, and presumably undergoes repeated rounds of abortive transcription prior to an onset of increased RNAPolII processivity in the early trophozoite stage, some 18–22 h after the invasion of the host erythrocyte [49]. The molecular basis of this increase in RNAPolII processivity remains to be determined, although conserved features of the T-regulatory loop and/or the heptad repeats within the carboxy-terminal domain of the *P. falciparum* RNAPolII large subunit would suggest regulation through kinase phosphorylation activity at these sites [50].

Placement of poly dA.dT tracts in the centre of NDR organised over *P. falciparum* promoters supports their role as intrinsic factors in determining nucleosome positioning in these regions. In budding yeast, poly dA.dT tracts have also been demonstrated to act as extrinsic factors in this same role by modulating interactions of promoter DNA with transcription factors, the RNAPolII complex and chromatin remodelling machinery to provide additional context, and directionality, for distinct promoter classes. The data presented here represent the average nucleosome positioning data, and whilst sufficient to demonstrate the intrinsic role, lacks context to explore any extrinsic role. That such a role may exist in *P. falciparum* is suggested by observed temporal variation in nucleosome positioning over promoters and the preferential positioning of non-canonical histone containing nucleosomes over IGR [14, 15, 51]. Resolution would require additional data correlating temporal gene expression patterns with binding of components of the transcriptional apparatus and/or chromatin remodelling machinery.

A key finding of this study was the highly overrepresented and overproportioned polydA.dT tracts in the IGR of the *Plasmodium spp*. compared to the other Apicomplexans investigated. Significantly, analysis of homopolymer tract organisation over the open reading frames in *Plasmodium spp*. reveals that polydA.dT are similarly highly overrepresented and overproportioned in these regions. This organisation of polydA.dT over open reading frames is quite distinct from the other Apicomplexans investigated here, and eukaryotic genomes in general [1, 3]. The presence of polyA tracts within *P. falciparum* open reading frames is well documented; leading to a pronounced codon usage bias and inclusion of low complexity amino acid stretches (particularly polylysine and polyasparagine) within the protein products. This same bias in codon usage is also evident throughout *Plasmodium spp*., similarly trending towards the inclusion of polyA tracts, although not to the extent of the extremely AT-rich *P. falciparum*. These low complexity regions (LCR) have been implicated in a variety of regulatory mechanisms including control of translational efficiency, a “smokescreen” of immunodominant regions assisting in the evasion of the host adaptive immune response, or as a protective response to heat-shock [52–56]. Reconciling these diverse adaptive and mechanistic roles for low complexity regions has recently been facilitated by their classification into three distinct groups based on their AT-content and heterozygosity [56]. Poly dA.dT would form part of the polyN group, exhibiting both high AT content and high levels of heterozygosity. No clear role for the polyN class of LCR exists, although speculation for a role in translational efficiency or an equally plausible neutral role has been made. For the neutral role, some form of selection pressure would be required to promote and/or retain these polyN LCR. This pressure may exist if we consider a role for poly dA.dT tracts in the formation of origins of replication in *Plasmodium spp*., as have been implicated in both budding and fission yeast [4, 6], during the critical process of infection and colonisation of the female mosquito following a blood meal on an infected vertebrate host. *Plasmodium spp*. are haploid for the majority of their life-cycle, with diploid stages only present in early stages of development in the mosquito midgut. Gametogenesis starts in erythrocytes and is completed when the macro- (female) and micro- (male) gametocytes are taken up during a mosquito blood-meal. The final maturation step for the microgametocyte, termed exflagellation, is a remarkable process taking some 20 minutes during which the microgametocyte undergoes three rounds of replication to generate eight flagellated microgametes [57–59]. This rapid replicative cycle would require each of the three rounds of DNA replication to take place over a 3–5 minute window, which, assuming a processivity of the order of 1-2kbp/min for eukaryotic replication [60], would require an exceptionally high density of origins of replication throughout the genome. As some 50% of *Plasmodium spp*. genomes encode protein, with a gene density of between 2.6-4.6 kb/gene, these origins of replication likely cannot be restricted solely to IGR, and may therefore provide a novel explanation to account for the significant overrepresentation and overproportionment of polydA.dT observed in both ORF and IGR of *Plasmodium spp*. genomes.

## Conclusion

Unlike previous comparative studies that emphasise evolutionary diversity in their determination of features of homopolymeric tract representation, length and spatial organisation, this study is instead restricted to a single phylum of unicellular parasites that all share moderately compact genomes. We describe features of polydA.dT tract organisation within this phylum that support a canonical role as intrinsic regulators of nucleosome positioning as well as findings that support nucleotide fraction as a key determinant in the thermodynamic threshold for tract expansion. Critically, we also present evidence for a novel lineage-specific organisation of homopolymer tract organisation in both intergenic and genic compartments along with evidence that overproportionment of homopolymer tract length may be dependent on the available intergenic space into which expand. Given the general lack of specific transcription factors in Apicomplexan genomes, a dynamic programme of nucleosome binding and rearrangement likely plays a significant role in the temporal and absolute regulation of transcription. Our observations relating to polydA.dT spatial arrangement and enrichment likely reflects the impact of chromatin structure and function in shaping the genomes of these parasites important for human and animal health.

## Methods

### Homopolymer tract frequency analysis using poly

Annotated genome sequences for *Plasmodium spp*. (PlasmoDB.org release 5.5), *Theileria spp*. and *B. bovis* (GeneDB.org), *Cryptosporidium spp*. (CryptoDB.org release 5.5) and *T. gondii* and *N. caninum* (ToxoDB.org release 5.1) were secured from their respective database repositories. Genbank-formatted documents were created that secured specified sizes of intergenic sequences that flank the coding sequence in each genome, taking up to the length specified unless a flanking coding sequence was encountered – in which case only intergenic sequence was secured in a truncated file. These sequence files were then concatenated into ASCII text files, one each for upstream and downstream flanking sequences. The ends of each individual flanking sequence were tagged to prevent the joining of sequences that may create a homopolymeric tract artefact between two sequences. Individual files were analysed using the program Poly [34], open source software publically available from http://www.bioinformatics.org/poly. Non-overlapping homopolymer tracts of all four types are counted by Poly for the entire file and a number of parameters are then calculated, including: the total base count for each file, its GC composition, and the numbers and the frequencies of the homopolymer tracts of different lengths. A moving window of 1 bp in length is used by Poly to differentiate tracts and spacers, taking into account the tags used to prevent artifactual sequence concatenation. These data and additional information are kept as data objects in the program and can be manipulated in various ways. Since Poly has been described in detail in previous reports [3, 34], we next only define its parameters that were used in this study.

The observed tract frequency of a given base i, *f* i_N_obs, of length N is determined in Poly by the formula: *f* i_N_obs = *c*i_N_obs/*l*_seq_.

where *c*i_N_obs is the count of observed tracts of base i at the specific length *N* contained in each sequence and *l*_seq_ is the total length of the sequence (total base count) in which those tracts were counted.

The expected frequency, *f* i_N_exp, of a homopolymer tract of base i of length N randomly occurring is calculated by the formula: *f* i_N_ exp = *f* i_N_obs^N^ × (1 ‒ *f* i_1_obs)^2^.

where *f* i_1_obs is the fractional base composition of that tract base.

The level of tract representation for a given base is then calculated as the ratio of observed to predicted frequencies, defined as Representation, *R*: *R* = *f* i_N_obs/ *f* i_N_exp.

Tracts are over-represented for *R* values larger than 1, and under-represented for values less than 1. The log(*R*) vs N plots were generated using GraphPad Prism v6.0 (GraphPad Software, La Jolla, USA). Linear interpolation of the data was used to determine the values of N at log*R* values of 0.5 (defined here as the threshold for over-representation). The slope of over-representation (slope_*R*_*)* was determined by the same linear interpolation of *R* between the threshold for over-representation and N_max_obs of each tract type. To reduce the noise in the analysis associated with infrequent observations of very long tracts, N_max_obs is scored only when *c*i_N_obs ≥ 4.

The maximum expected length of a homopolymer tract of any base N_max_exp, is calculated for any sequence length based upon random base sequence occurrence in a sequence of that length of equivalent base composition to the real sequence. The maximum homopolymer tract length observed in the real sequence, N_max_obs*,* can then be compared to its expected length by taking the following ratio, a parameter defined as proportion*, P* = N_max_obs*/*N_max_exp.

The condition of overproportionment is defined for *P* values larger than 1, while underproportionment is for *P* values less than 1.

### Calculating tract frequencies as a function of sequence position

We developed a non-overlapping motif frequency counting program called motif.freq.pl., written in perl v.5.12.4., which can be accessed at http://www.bioinformatics.org/motiffreq. It calculates the individual bin frequencies of any specified input sequence motif within a binned sequence. Motif.freq.pl requires the user to enter the input sequence motif to be searched and the bin size. The input sequence(s) is divided evenly into sequence bins of size determined by the input bin size. The program will count the occurrences of the input sequence motif within each bin and return the values to the output file as a motif frequency defined as F_obs_, the number of motif occurrences/nucleotides in the bin. If the motif is repeated in the sequence without interruption by intervening bases, motif.freq.pl will count as many motifs as the program can detect. If the motif is physically located at the bin interface and occurs within two adjacent bins, the motif will be counted as being within the bin which contains the most bases of the motif. If the motif contains an equal number of bases located within two adjacent bins, the motif will be considered to reside within the first bin being counted in the sequence. The output of motif.freq.pl is a text file listing the bin number and motif frequency for each bin.

We also created a sequence-shuffling program that conserves the original input sequence’s base frequencies during shuffling. This program is called shuffle.pl, also written in perl v.5.12.4., and it can be accessed at http://www.bioinformatics.org/motiffreq. The purpose of this program was to assess, by comparison, the significance of the motif.freq.pl program’s frequency-bin output resulting from the input of real sequences. The shuffling program reads multiple sequences of specified length in FASTA format and then merges all sequences together into one master combined string. The program then shuffles all bases in the combined string, using the shuffle function in the LIST::Util module in PERL. The shuffled master string is then broken up into the original number and length of sequences used as input. These shuffled sequences were then used as input to motif.freq.pl, producing output to allow exact comparison of the original sequence’s frequency-bin distribution to the shuffled sequence’s to assess for locations of motif enrichment or depletion. Shuffle.pl allows the user to specify the number of times that the motif shuffling is to be applied to the original input sequences. Thus, for a large number of shufflings and by averaging the shuffled frequencies in equivalent bins, a much lower ‘noise’ or variation in the shuffled random frequency-bin distribution can be achieved to compare to the original sequence motif bin distribution. As a test, real sequences were input into the shuffle.pl program to generate ten-fold randomly shuffled sequences. For a number of calculated short sequence motif frequency-bin distributions, these control shuffled distributions all exhibited frequency vs bin # plots with low variation between bins. The linear fits to these plots possessed slopes near zero (R^2^ < 0.05), indicating no statistically significant trend in the shuffled sequential bin frequencies, as expected. In this study, we carried out 10 shufflings of each real sequence analyzed and used these as input to motif.freq.pl. The 10 motif.freq.pl output sequences’ frequencies were then averaged for each bin to create an average random frequency bin distribution to compare to each real sequence. We call this frequency average of 10 shufflings: F_10X shuffle_.

## Electronic supplementary material

Additional file 1: **Supplementary Figures and Tables.** Contains Figures S1-S6 and Tables S1 and S2. (PDF 3 MB)

## Abbreviations

EGASP: ENCODE Genome Annotation Assessment Project
fiNexp: Frequency (nucleotide i and length N) expected
fiNobs: Frequency (nucleotide i and length N) observed
Fobs: Frequency observed
F10X shuffle: Frequency following a 10-fold shuffle
FAIRE: Formaldehyde-assisted isolation of protein-free DNA
IGR: Intergenic regions
LCR: Low complexity region
MAINE: Micrococcal Nuclease-assisted isolation of nucleosome bound DNA
NDR: Nucleosome depleted region
Nmaxexp: Maximum length of tract expected
Nmaxobs: Maximum length of tract observed
ORF: Open reading frame
P: Proportion
PIC: Pre-initiation complex
R: Representation
RNAPolII: RNA polymerase II complex
Type A IGR: An IGR flanked with head-to-head genes containing two promoters
Type C IGR: An IGR flanked with tail-to-tail genes containing two terminators.
