# Supplementary material for: Homopolymer tract organization in the human malarial parasite Plasmodium falciparum and related Apicomplexan parasites
Source: BMC Genomics. 2014 Oct 3;15(1):848. doi: 10.1186/1471-2164-15-848 (PMC4194402; doi:10.1186/1471-2164-15-848)
Supplement: Supplementary file 1 — Additional file 1: Supplementary Figures and Tables. Contains Figures S1-S6 and Tables S1 and S2. (PDF 3 MB) [file 12864_2014_6522_MOESM1_ESM.pdf]

Additional File 1

Homopolymer tract organization in the human malarial parasite *Plasmodium falciparum* and related Apicomplexan parasites.

Karen Russell[1], Chia-Ho Cheng[2,8], Jeffrey W. Bizzaro[3], Nadia Ponts[4], Richard D. Emes[5,6], Karine Le Roch[7], Kenneth A. Marx[2] and Paul Horrocks[1,*]

[1]Institute for Science and Technology in Medicine, Keele University, Staffordshire ST5 5BG, United Kingdom; [2]Center for Intelligent Biomaterials, University of Massachusetts Lowell, MA 01854, USA; [3]Bioinformatics Organization Inc., Hudson, MA 01749, USA; [4]National Institute for Agricultural Research (INRA), UR1264-Mycology and Food Safety (MycSA), CS20032, 33882 Villenave d'Ornon Cedex, France; [5]School of Veterinary Medicine and Science, University of Nottingham, Leicestershire LE12 5RD, United Kingdom. [6]Advanced Data Analysis Centre, University of Nottingham UK. [7]Dept. Cell Biology and Neuroscience, University of California, Riverside, CA 92521, USA. [8] Currently at Institute for Aging Research, Hebrew SeniorLife, Boston, MA 02131, USA

Supplementary Figures S1 to S6

Supplementary Tables 1 and 2

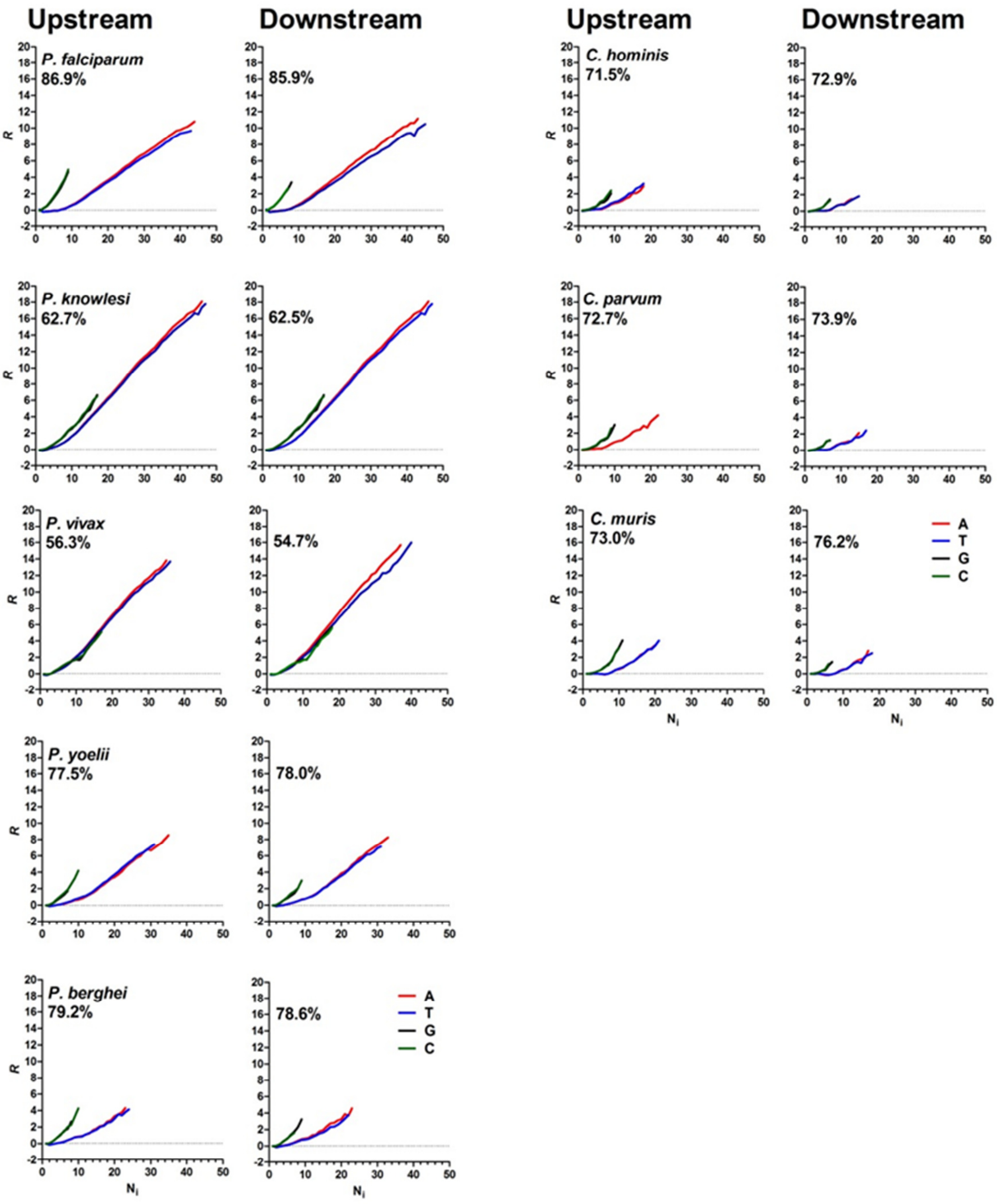

Fig. S1. Representation of homopolymeric tracts in the proximal upstream and downstream intergenic flanking regions of *Plasmodium spp*. and *Cryptosporidium spp*. (above) These graphs plot *R* for homopolymer tracts (see key, lower right) as a function of their length (N_i) for those organisms where short poly dG.dC and long poly dA.dT tracks are overrepresented. The %AT content of the proximal upstream and downstream intergenic sequences analysed is reported on each graph.

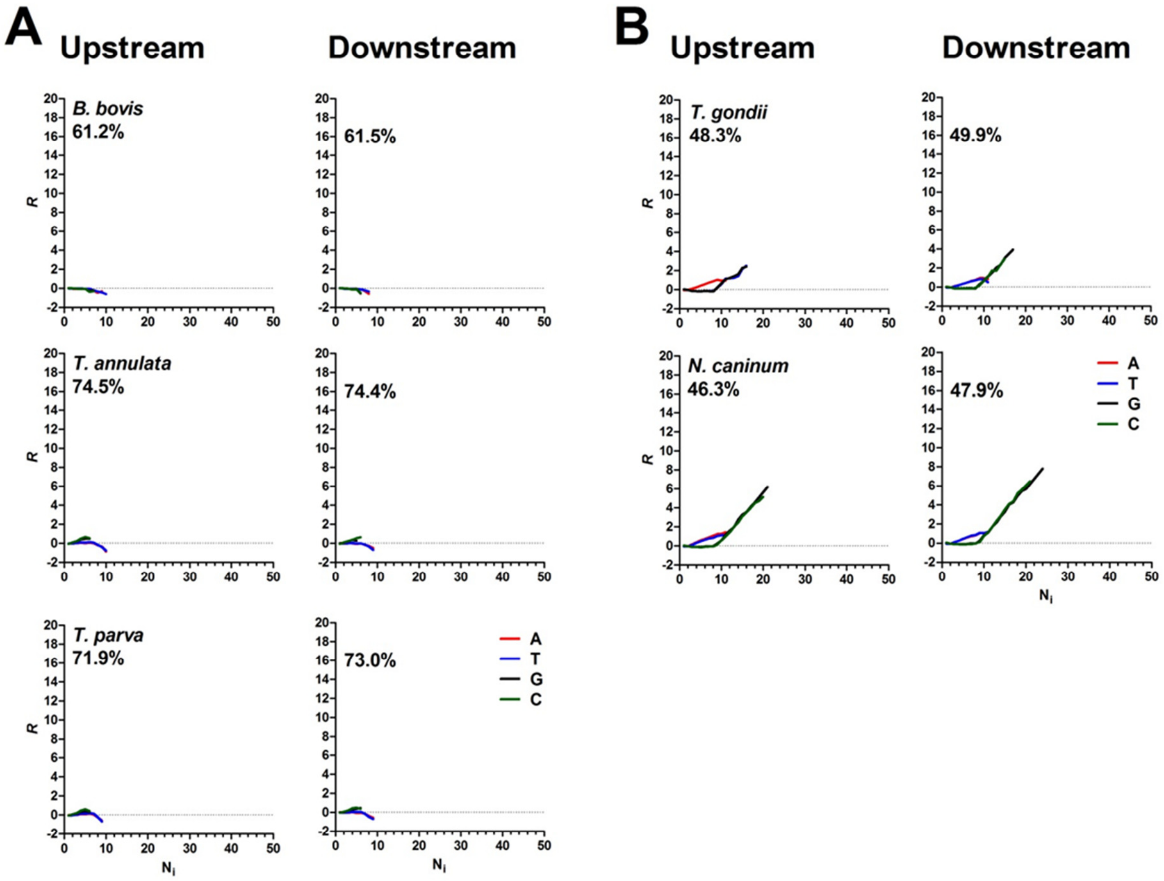

Fig. S2. Representation of homopolymeric tracts in the proximal upstream and downstream intergenic flanking regions of the coccidian and piroplasmida organisms (above). These graphs plot *R* for homopolymer tracts (see key, lower right) as a function of their length (N_i) for those organisms where either (**A**) there was no evidence for overrepresentation of any homopolymeric tracts (*Theileria spp*. and *B. bovis*) or (**B**) long poly dG.dC and short poly dA.dT tracks are overrepresented. The %AT content of the proximal upstream and downstream intergenic sequences analysed is reported on each graph.

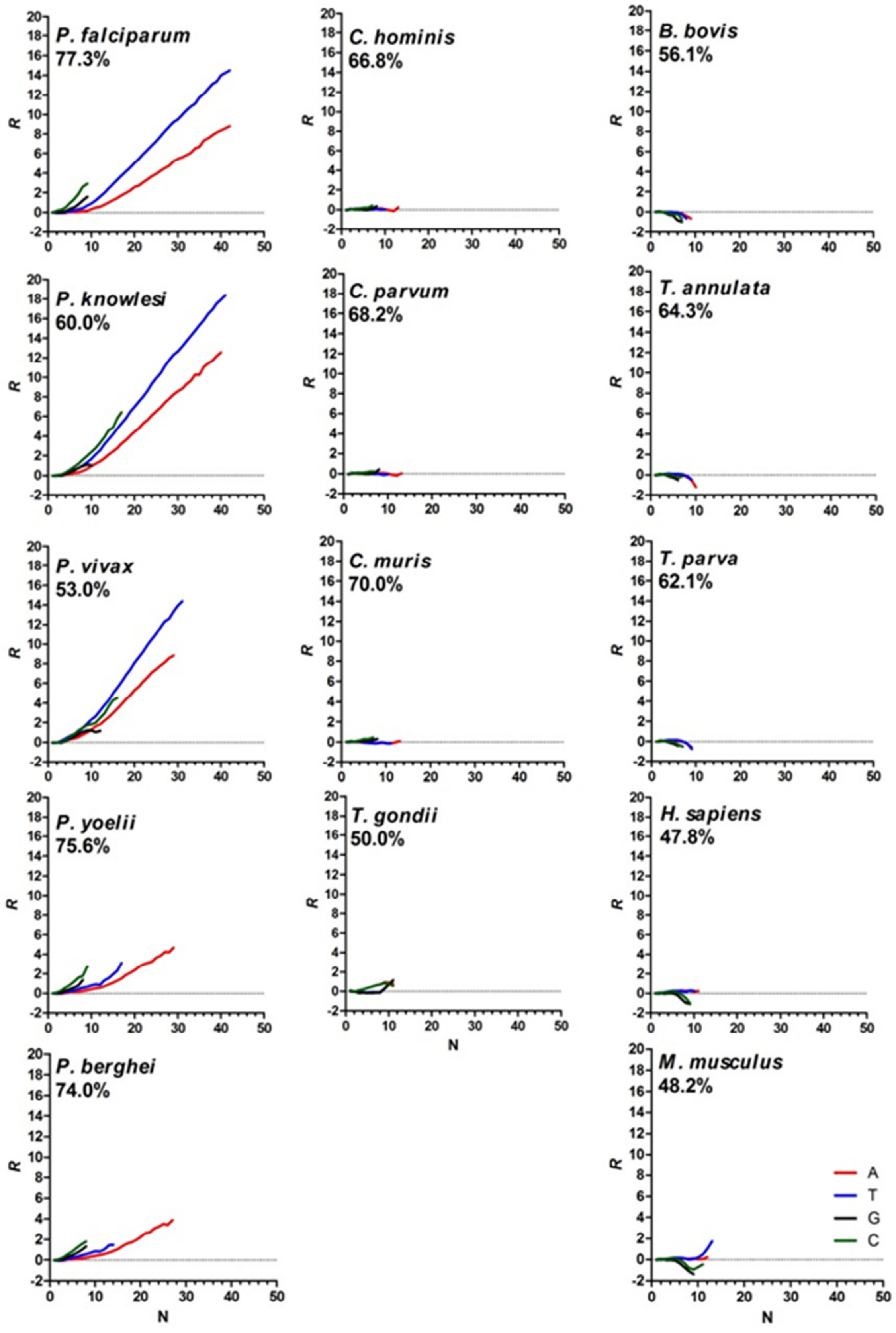

Fig. S3. Representation of homopolymeric tracts over coding sequences (above). These graphs plot *R* for homopolymer tracts (see key, lower right) as a function of their length (N_i). The species as well as the average %AT content of the coding sequences analysed is reported on each graph.

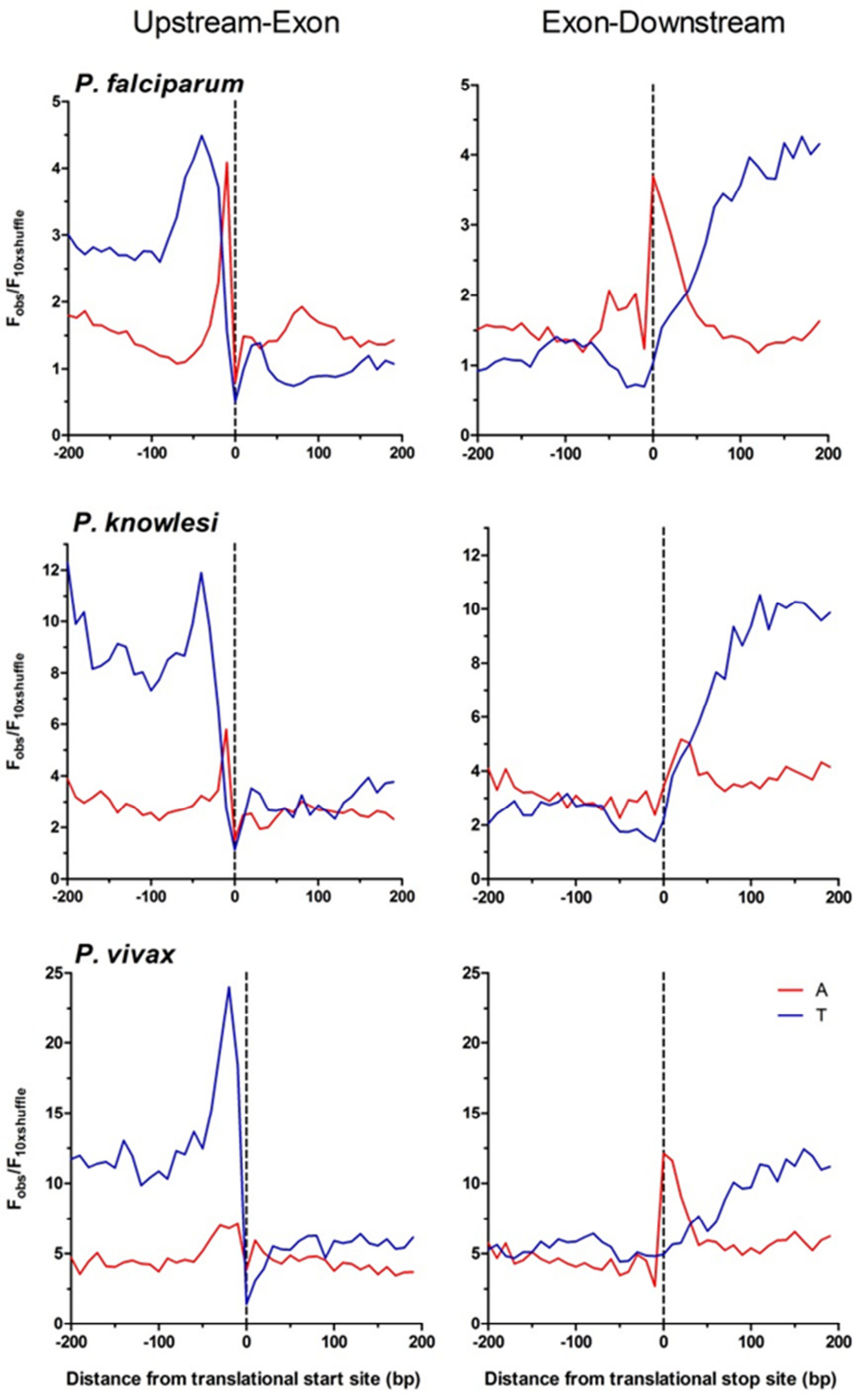

Fig. S4. Relative spatial distribution of poly dA.dT tracts over translational start and stop sites of ORF from three *Plasmodium spp*. (above) Plots are presented of the spatial distribution (bin size of 10 bases, x-axis) of relative frequency of poly dA (red line) and poly dT (blue line) tracts of 5 base length in the 200 bases of sense strand flanking either side of the translational start (upstream-exon) and stop (exon-downstream) sites. The Y-axis reports the relative frequency of observed (F_{obs}) non-overlapping tracts divided by the frequency of the same tracts from a 10 x random shuffled average of the same sequences (i.e. same base composition retained in the 10X shuffle, $F_{10xshuffe}$) to normalise F_{obs} across the diverse range

of nucleotide content in these *Plasmodium spp*. Note, compared to Fig. 7 the relative frequency of these peaks increases as the AT content decreases from *P. falciparum* to *P. knowlesi* and *P. vivax*.

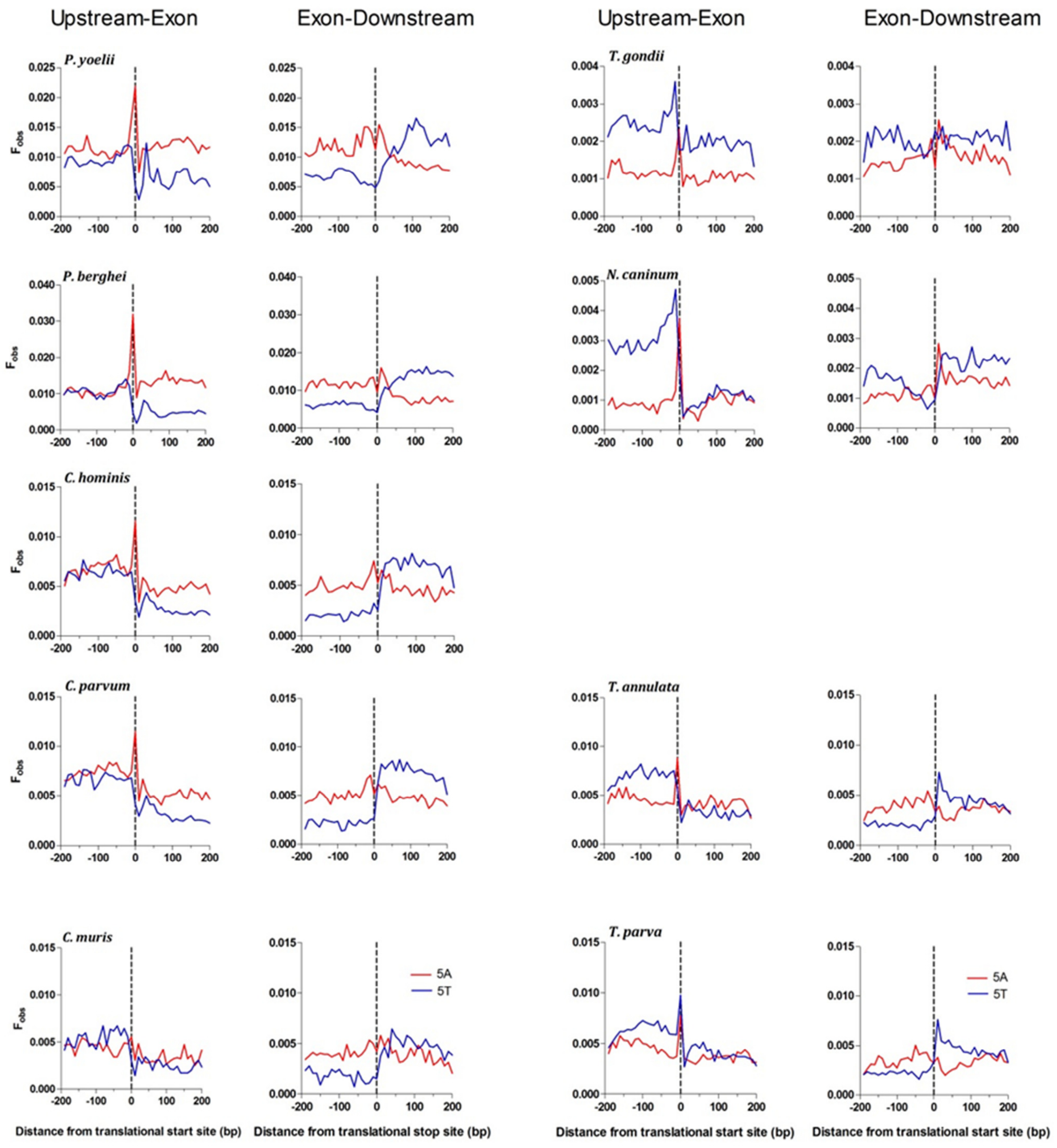

Fig. S5 Enrichment of poly dA.dT tracts proximal to translational start and stop sites of ORF is a typical feature of intergenic flanking sequences in the organisms used in this study (above). Plots of the spatial distribution (bin size of 10 bases, X-axis) of frequency (F_{obs}) of poly dA (red line) and poly dT (blue line) tracts of 5 base length in the 200 bases of sense strand flanking either side of the translational start (upstream-exon) and stop (exon-downstream) sites.

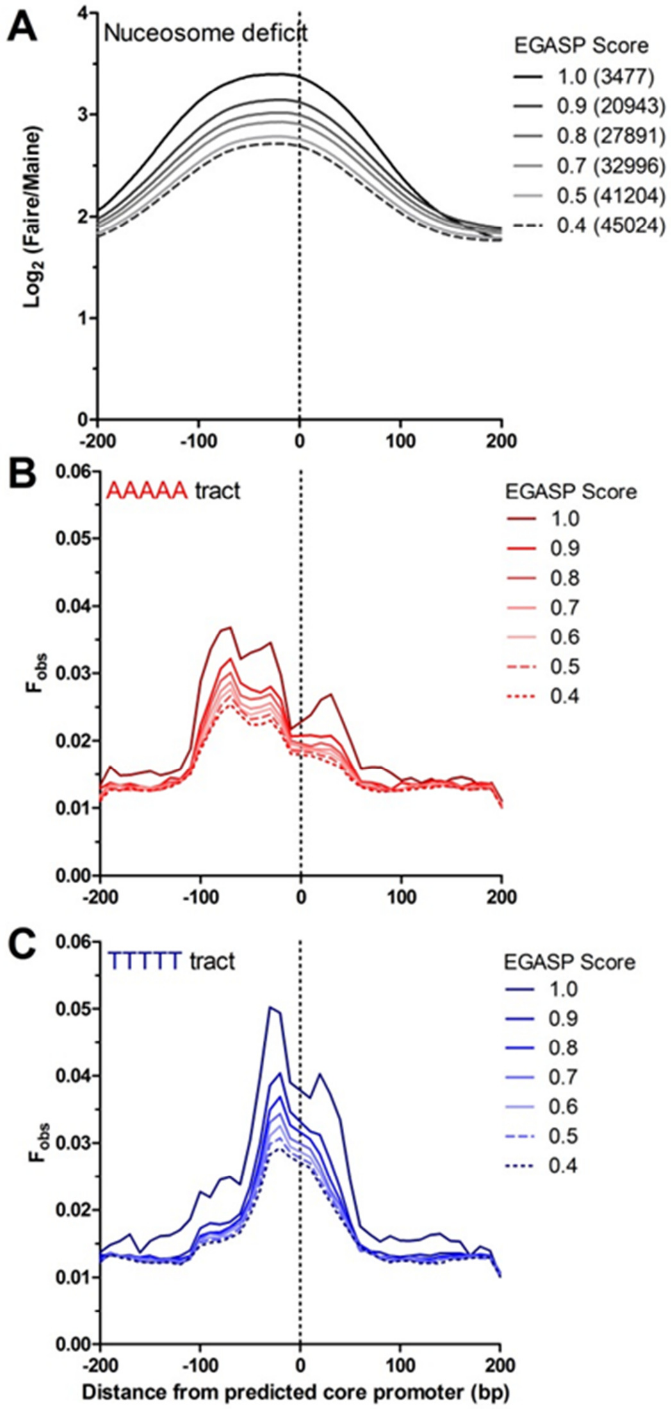

Fig. S6. Spatial distribution of nucleosome occupancy and poly dA.dT tracts over *P. falciparum* predicted core promoters of varying confidence (above). Prediction of core promoter regions as reported using the Malarial Promoter Predictor (MAPP) tool, derives scores that can be clustered based on their positive predictive value and sensitivity (using EGASP criteria, where 0.4 is moderately confident and 1 is highly confident). The 200 bases of sense strand sequence flanking either side of predicted core promoters for EGASP thresholds 0.4 to 1 were analysed to report (**A**) the relative nucleosome deficit over these core promoters (log_2 FAIRE/MAINE sequence reads), with the key reporting the number of core promoters analysed in each case in parentheses, and the F_{obs} of N=5 tract lengths of poly dA (**B**) and poly dT (**C**).

Organism	Family	% AT[1]	Gene Density[2]	Median size of IGR[3] (bp)			Ratio of IGR size[4]			Ups (bp)[5]	Down (bp)[5]
				A	B	C	A	B	C		
Plasmodium falciparum	Plasmodiidae	80.6	4.3	1938	1385	677	2.9	2.0	1.0	2000	700
Plasmodium knowlesi	Plasmodiidae	62.5	4.6	2162	1592	736	2.9	2.2	1.0	2000	700
Plasmodium vivax	Plasmodiidae	57.7	4.5	1956	1434	643	3.0	2.2	1.0	2000	700
Plasmodium yoelli	Plasmodiidae	77.4	2.6	1192	578	582	2.0	1.0	1.0	1000	700
Plasmodium berghei	Plasmodiidae	79.2	3.1	na	na	na	na	na	na	1000	700
Cryptosporidium hominis	Cryptosporidiidae	68.3	2.3	640	494	203	3.2	2.4	1.0	650	200
Cryptosporidium parvum	Cryptosporidiidae	70.0	2.4	634	460	175	3.6	2.6	1.0	650	200
Cryptosporidium muris	Cryptosporidiidae	83.0	na	na	na	na	na	na	na	650	200
Toxoplasma gondii	Sarcocystidae	47.7	9.1	2576	2437	1623	1.6	1.5	1.0	2500	1600
Neospora caninum	Sarcocystidae	45.2	8.6	3603	3899	2172	1.7	1.8	1.0	2500	1600
Babesia bovis	Babesiidae	58.2	2.2	543	352	175	3.1	2.0	1.0	550	200
Theileria annulata	Theileriidae	67.5	2.2	439	277	125	3.5	2.2	1.0	450	150
Theileria parva	Theileriidae	65.9	2.1	376	256	154	2.4	1.7	1.0	450	150

[1] %AT content of whole genome. [2] As total genome/ number of genes in kbp. [3] InterGenic Region. [4] Where size of IGR C is defined at 1. [5] Length of proximal upstream (Ups) and downstream (Down) sequence investigated here.

Table S1

Organism	Fraction$_l$ frequency base				Maximum length observed N_{max}obs				Maximun length expected M_{max}exp				Proportionment (P)				Threshold (R >0.5)				Slope$_R$ [1]			
	A	C	G	T	A	C	G	T	A	C	G	T	A	C	G	T	A	C	G	T	A	C	G	T
P. falciparum	0.45	0.09	0.13	0.32	42	9	9	42	20	6	8	14	2.10	1.50	1.13	3.00	12	4	6	9	0.28	0.50	0.36	0.44
P. knowlesi	0.35	0.18	0.22	0.25	40	17	10	42	15	9	10	11	2.67	1.89	1.00	3.82	9	5	6	6	0.40	0.48	0.21	0.54
P. vivax	0.31	0.22	0.25	0.22	29	16	12	32	13	10	11	10	2.23	1.60	1.09	3.20	7	5	6	5	0.40	0.36	0.23	0.56
P. yoelii	0.44	0.10	0.14	0.32	29	7	8	17	19	6	7	13	1.53	1.17	1.14	1.31	11	4	6	8	0.24	0.39	0.37	0.26
P. berghei	0.45	0.10	0.14	0.31	24	7	9	22	19	7	7	13	1.26	1.00	1.29	1.69	12	4	4	8	0.22	0.30	0.32	0.15
C. hominis	0.36	0.15	0.18	0.31	13	7	8	10	14	8	8	12	0.93	0.88	1.00	0.83	na	na	na	na	na	na	na	na
C. parvum	0.37	0.14	0.17	0.31	13	9	8	10	15	8	8	13	0.87	1.13	1.00	0.77	na	na	na	na	na	na	na	na
C. muris	0.37	0.14	0.16	0.33	13	7	8	11	16	7	8	14	0.81	1.00	1.00	0.79	na	na	na	na	na	na	na	na
T. gondii	0.25	0.25	0.25	0.25	11	15	15	12	11	11	11	11	1.00	1.36	1.36	1.09	7	10	10	7	0.05	0.41	0.43	<0.01
B. bovis	0.29	0.21	0.23	0.27	8	6	6	8	12	9	10	11	0.67	0.67	0.60	0.73	na	na	na	na	na	na	na	na
T. annulata	0.35	0.17	0.19	0.29	9	6	5	9	14	8	9	12	0.64	0.75	0.56	0.75	na	na	na	na	na	na	na	na
T. parva	0.34	0.18	0.20	0.28	9	6	6	9	14	9	9	12	0.64	0.67	0.67	0.75	na	na	na	na	na	na	na	na
H. sapiens	0.26	0.26	0.26	0.22	11	9	9	10	12	12	13	11	0.92	0.75	0.69	0.91	na	na	na	na	na	na	na	na
M. musculus	0.26	0.26	0.26	0.22	12	11	9	13	13	13	13	11	0.92	0.85	0.69	1.18	na	na	na	12	na	na	na	0.73

[1]slope of R between threshold of overrepresentation and N_{max}obs. [2]na, not available

Table S2
